# Causal relationship between atrial fibrillation and leukocyte telomere length: A two sample, bidirectional Mendelian randomization study

**DOI:** 10.3389/fcvm.2023.1093255

**Published:** 2023-02-15

**Authors:** Zimo Sha, Tianzhichao Hou, Taojie Zhou, Yang Dai, Yangyang Bao, Qi Jin, Jing Ye, Yiming Lu, Liqun Wu

**Affiliations:** ^1^Department of Cardiology, Ruijin Hospital, Shanghai Jiao Tong University School of Medicine, Shanghai, China; ^2^Department of Endocrine and Metabolic Diseases, Shanghai Institute of Endocrine and Metabolic Diseases, Ruijin Hospital, Shanghai Jiao Tong University School of Medicine, Shanghai, China; ^3^Institute of Cardiovascular Diseases, Shanghai Jiao Tong University School of Medicine, Shanghai, China; ^4^Department of Geriatrics, Medical Center on Aging of Shanghai Ruijin Hospital, Shanghai Jiaotong University School of Medicine, Shanghai, China; ^5^International Laboratory in Hematology and Cancer, Shanghai Jiao Tong University School of Medicine/Ruijin Hospital/CNRS/Inserm/Côte d'Azur University, Shanghai, China

**Keywords:** bidirectional Mendelian randomization (MR), atrial fibrillation, genome-wide association study, leukocyte telomere length, expression quantitative trait loci (eQTL), protein quantitative trait loci (pQTL)

## Abstract

**Background:**

Atrial fibrillation (AF) is an age-related disease, while telomeres play a central role in aging. But the relationship between AF and telomere length (LTL) is still controversial. This study aims to examine the potential causal association between AF and LTL by using Mendelian randomization (MR).

**Methods:**

Bidirectional two-sample MR, expression and protein quantitative trait loci (eQTL and pQTL)-based MR were performed using genetic variants from United Kingdom Biobank, FinnGen, and a meta-analysis study, which comprised nearly 1 million participants in the Atrial Fibrillation Study and 470,000 participants in the Telomere Length Study. Apart from the inverse variance weighted (IVW) approach as the main MR analysis, complementary analysis approaches and sensitivity analysis were applied.

**Results:**

The forward MR revealed a significant causal estimate for the genetically predicted AF with LTL shortening [IVW: odds ratio (OR) = 0.989, *p* = 0.007; eQTL-IVW: OR = 0.988, *p* = 0.005; pQTL-IVW: OR = 0.975, *p* < 0.005]. But in the reverse MR analysis, genetically predicted LTL has no significant correlation with AF (IVW: OR = 0.995, *p* = 0.916; eQTL-IVW: OR = 0.999, *p* = 0.995; pQTL-IVW: OR = 1.055, *p* = 0.570). The FinnGen replication data yielded similar findings. Sensitivity analysis ensured the stability of the results.

**Conclusion:**

The presence of AF leads to LTL shortening rather than the other way around. Aggressive intervention for AF may delay the telomere attrition.

## 1. Introduction

Atrial fibrillation (AF) is a supraventricular tachyarrhythmia with uncoordinated atrial electrical activation and ineffective atrial contractions (1). As the most common type of clinical arrhythmia, AF involves 37.6 million patients globally and is expected to increase by more than 60% over the next 30 years (2, 3). However, the treatment of atrial fibrillation is currently limited to patients with severe symptoms or the prevention of its complications.

AF exhibits an increasing prevalence with chronological aging, while the cardiac remodeling and hemodynamic changes caused by AF in turn accelerate biological aging (4). The telomere, the cap-like protective structure at the end of the chromosome, is one of the major determinants of biological aging (5). Prematurely shortened leukocyte telomere length (LTL) has been confirmed to be related to a variety of risk factors of AF, including body mass index (BMI), obstructive sleep apnea syndrome, and diabetes mellitus (6, 7). However, whether there is a relationship between LTL and AF is still controversial. Several cross-section studies have shown the positive link between LTL and the occurrence of AF that age-adjusted LTL decreased by 9% for subjects with a history of AF versus controls (*n* = 3,576, *p* = 0.017) (8, 9). Short-term cohort studies also indicate that shorter LTL was associated with the progression and recurrence of AF (10–12). But the Framingham Heart Study and a meta-analysis revealed no association between LTL and AF (13, 14). A prospective study even suggested LTL tended to be longer in male individuals with AF compared to control (14). The inconsistent results of these previous observational studies may be due to the reverse causation or confounding bias between LTL and AF.

Mendelian randomization (MR) has emerged as a reliable method to address some limitations of observational studies which are minimally affected by reverse causation or confounding effects because of the fixed instrumented genetic information since birth (15). In the MR analysis, genetic variants closely associated with the exposure were considered instrumental variables (IVs) and used to infer a causal relationship between the exposure and the outcome.

**Figure 1:**
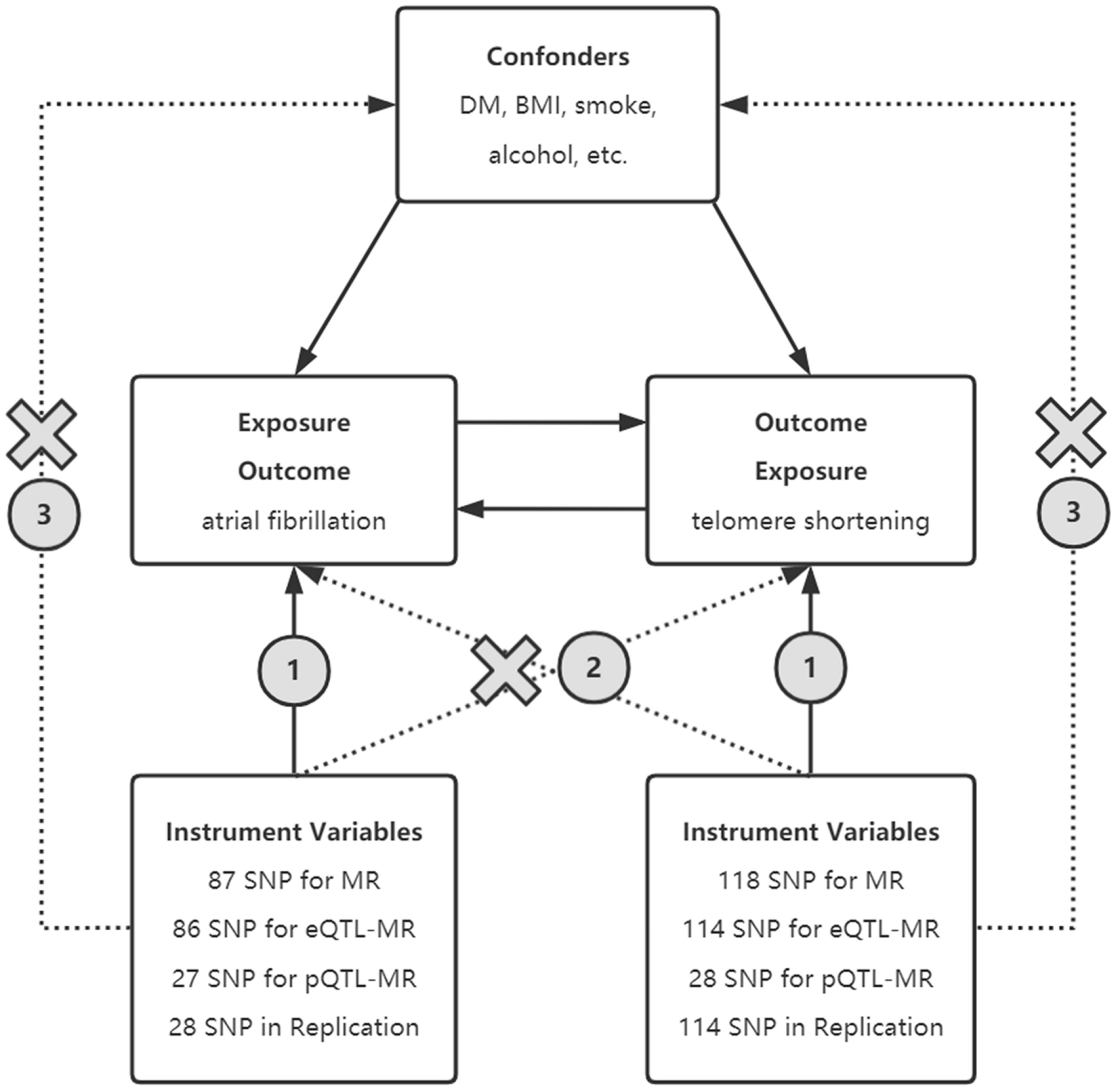
Principle of bidirectional Mendelian randomization. Selected SNPs as instrument variables in MR, eQTL-MR, and pQTL-MR analyses are strongly associated with exposure. The instrumental variables must be independent of the confounding factors and should only influence the outcome through exposure rather than other means. SNPs, single nucleotide polymorphism; BMI, body mass index; UKB, United Kingdom Biobank; eQTL, expression quantitative trait loci; pQTL, protein quantitative trait loci.

Here, a bidirectional, two-sample MR analysis was performed to explore the bidirectional causal linkage between AF and LTL. Furthermore, single-nucleotide polymorphisms (SNPs) associated with messenger RNA expression or protein levels, also referred to as expression or protein quantitative trait loci (eQTLs or pQTLs, respectively), were used as instruments for eQTL and pQTL-based MR. In the present study, we hypothesized that AF promotes telomere shortening and that short telomeres also contribute to the development of AF (Figure 1).

## 2. Materials and methods

### 2.1. Study design

The causal relationship was performed by bidirectional MR analysis using AF-associated genetic variants and LTL-associated genetic variants as IVs in forward and reverse MR, respectively. eQTL-based, pQTL-based, and replication MR was applied to ensure the stability of the results (Figure 1). This analysis was reported according to the STROBE-MR guidelines (16).

### 2.2. Data source

The summary statistic data for AF in original analysis was derived from a GWAS meta-analysis of six studies including the Nord-Trøndelag Health Study, deCODE, the Michigan Genomics Initiativ, DiscovEHR, United Kingdom Biobank, and the AFGen Consortium, which totally enrolled 60,620 AF cases and 970,216 controls of European ancestry (17). Data for AF in replication analysis contains 22,068 AF cases and 196,724 controls deriving from the FinnGen cohort, which is a large public-private partnership aiming to collect and analyze genome and health data from 500,000 Finnish biobank participants. Meanwhile, genetic variants associated with LTL were selected from the United Kingdom Biobank, which is one of the largest biomedical databases collecting genetic and health data of 500,000 United Kingdom individuals and conducting critical research on the most common prevalent or fatal diseases. The LTL was measured by an established quantitative PCR assay in 472,174 United Kingdom Biobank participants of European ancestry (18).

### 2.3. Instrumental variable selection

The extracted genetic variants were featured as IVs to estimate the bidirectional causal effects between LTL and the risk of AF based on the following assumptions: (1) the IVs are strongly associated with AF (or LTL in the reverse-direction analysis), (2) the IVs are not associated with confounders between AF and LTL, and (3) no alternative outcome through independent pathways apart from AF (or LTL in the reverse-direction analysis) (19).

SNPs associated with AF and LTL at a genome-wide significance (*p* < 5 × 10^−8^) were included. After pruning for linkage disequilibrium (LD; *r*^2^ < 0.001; distance <10,000 kb), each of the AF-associated and LTL-associated SNPs was checked for potential violations of assumption 2 and 3 *via* the Phenoscanner database (20, 21). We excluded SNPs that were significantly (*p* < 1 × 10^−4^) associated with any known confounders between AF and LTL (e.g., alcohol intake, smoking, BMI, inflammation, and Diabetes mellitus) (22, 23). Those who were not included in the outcome GWAS or were defined as incompatible or palindromic SNPs with intermediate allele frequencies will be withdrawn. The eQTL and pQTL SNPs were generated by analyzing SNPs in the Phenoscanner database at a genome-wide significance (*p* < 5 × 10^−8^ and *p* < 1 × 10^−4^, respectively) (24–30).

### 2.4. Statistical analysis

#### 2.4.1. Mendelian randomization analyses

In this bidirectional MR study, we performed a random-effect inverse variance weighting (IVW) method as the primary analysis to assess the bidirectional causal effect between LTL and AF, which can reach higher estimation accuracy and test power when the IVs satisfy the three basic assumptions (31). Additional MR methods were applied to lighten the confounding presence of horizontal pleiotropy, heterogeneity, and violation of underlying assumptions, including MR-Egger regression, Weighted median, simple mode, and weighted mode (32). A robust adjusted profile score (RAPS) estimator was also used to adjust the contour scores to improve the robustness and efficiency of the assessment (33). Considering the possible sample overlap in the original analysis, we used Maximum likelihood analysis to reduce the effect of weak instrumental bias on the results.

#### 2.4.2. Sensitivity analyses

We calculated the *R* statistics of the selected LTL-associated SNPs and AF-associated SNPs to detect the proportion of variation explained by the SNPs in the exposure. The *F* statistics were estimated as $F=\frac{R^{2}}{1-R^{2}}\times \frac{N-1-k}{k}$ , which *k* represents the number of IVs and *N* represents the sample size in the exposure data. The weak instrumental bias can be ignored when the *F* statistic >10.

We assessed heterogeneity between SNPs within LTL-associated and AF-associated IVs using IVW and MR-Egger estimates (34). Leave-one-out analysis was additionally performed to identify if a single SNP is driving the caution association between the exposure and the outcome (32). We evaluated horizontal pleiotropy in this bi-directional MR study. When the IVs affect the outcome outside of their effect on the exposure, horizontal pleiotropy will occur. We used the Mendelian randomization pleiotropy residual sum and outlier (MR-PRESSO) test to examine the horizontal pleiotropy and to compare the causal estimates from the IVW analysis after removing outlier IVs (35).

All analyses in this study were performed in R software (Version 4.1.3) by the R package (TwoSampleMR, MRPERSSO, mr.raps) (32, 36). Results were presented as odds ratios (OR) together with the 95% confidence interval (CI). A two-sided value of p less than 0.05 was considered statistically significant.

## 3. Results

### 3.1. Selection of instrumental variables (original analyses)

In the bidirectional MR analyses, we obtained 111 and 154 linkage disequilibrium-independent SNPs for AF and LTL, respectively. Among these SNPs, 9 AF-associated SNPs and 11 LTL-associated SNPs were not included in the outcome database. One AF-associated and 4 LTL-associated incompatible or palindromic SNPs with intermediate allele frequencies were also withdrawn. Additionally, 14 and 21 SNPs were eliminated for possible infractions of assumptions 2 and 3 *via* the Phenoscanner database. In total, we applied 87 SNPs as instrumental variables for AF and 118 SNPs for LTL, explaining 0.8% (*R*^2^) of the AF variation and 3.0% (*R*^2^) of the LTL variation. The F-statistics calculated from the forward and the reverse MR regression model were 46 and 64, respectively. Of the 87 AF-SNPs, 86 were thought to be eQTL-SNPs related with mRNA expression and 27 were thought to be pQTL-SNPs connected with protein expression. Of the 118 LTL-SNPs, 114 associated with mRNA expression were regarded as eQTL-SNPs, and 28 associated with protein expression were regarded as pQTL-SNPs. Supplementary Tables S1, S2 provided comprehensive details on the traits of AF-associated SNPs and LTL-associated SNPs.

### 3.2. Selection of instrumental variables (replication analyses)

According to the same method as above, we applied 28 SNPs for AF in the forward replication analyses and 114 SNPs for LTL in the reverse replication analyses. The Supplementary Tables S3, S4 have detailed description of each.

### 3.3. Causal association of AF with LTL *via* forward MR (original analyses)

**Figure 2:**
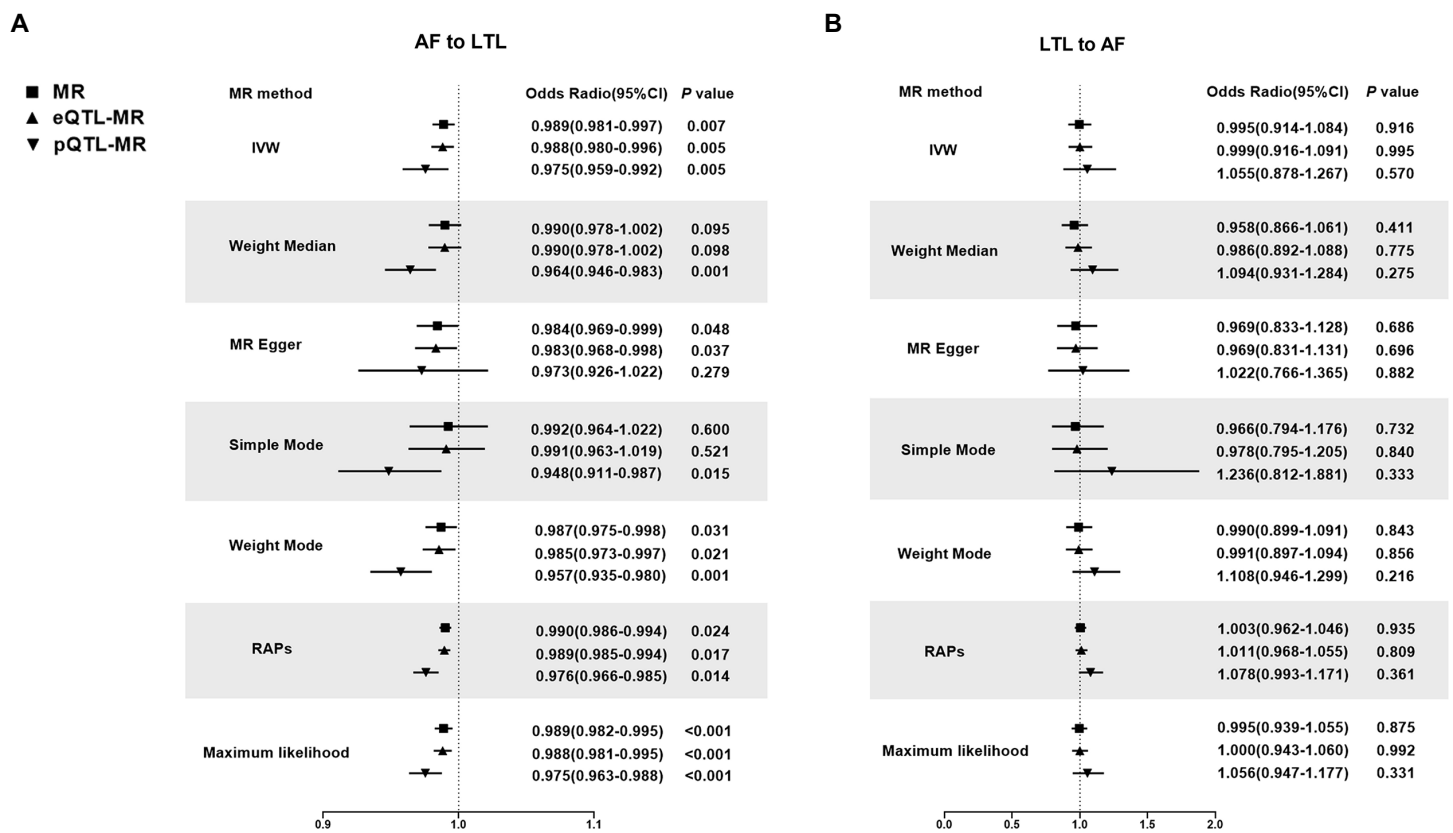
Mendelian Randomization estimators of the causal relationship between leucocyte telomere length and atrial fibrillation in original analyses. **(A)** AF-LTL. **(B)** LTL-AF. Analyses were conducted using the conventional IVW, Weighted median, MR Egger, Simple mode, Weighted mode, Robust adjusted profile score (RAPS) methods, Maximum likelihood. The slope of each line corresponds to the estimated MR effect per method. MR, Mendelian randomization; IVW, Inverse variance weighted; RAPS, robust adjusted profile score; LTL, leucocyte telomere length; AF, atrial fibrillation; eQTL, expression quantitative trait loci; pQTL, protein quantitative trait loci.

**Figure 3:**
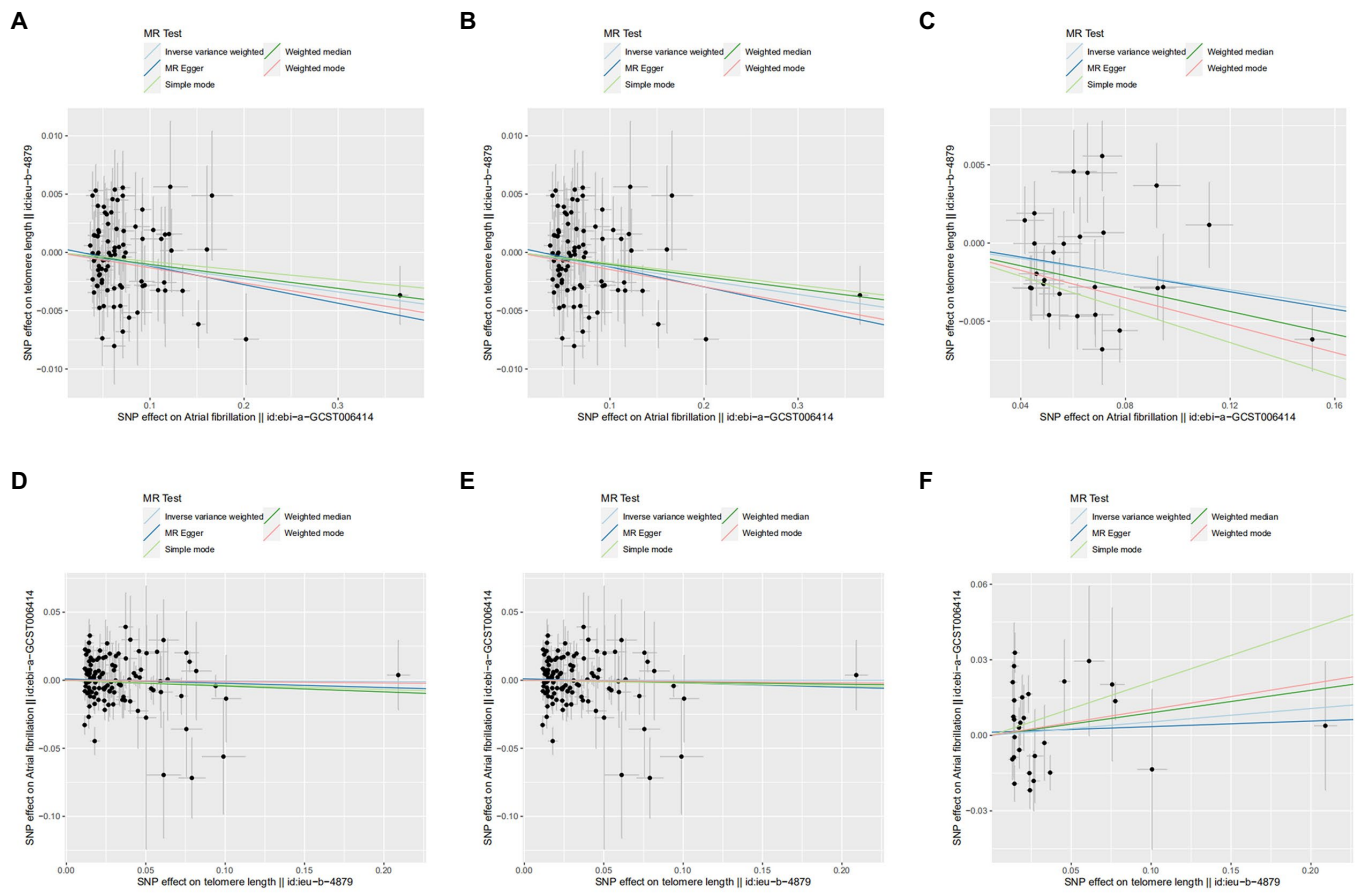
Scatter plot of SNPs associated with leucocyte telomere length and atrial fibrillation. **(A)** AF-LTL in MR analysis. **(B)** AF-LTL in eQTL-MR analysis. **(C)** AF-LTL in pQTL-MR analysis. **(D)** LTL-AF in MR analysis. **(E)** LTL-AF in eQTL-MR analysis. **(F)** LTL-AF in pQTL-MR analysis. The plot related the effect sizes of the SNP–exposure association (*x*-axis, SD units) and the SNP–outcome associations [*y*-axis, log (OR)] with 95% confidence intervals. The regression slopes of the lines correspond to causal estimates based on the inverse variance weighted method, MR-Egger regression, weighted median, simple mode, and weighted mode estimator. MR, Mendelian randomization; SNP, single-nucleotide polymorphism.

MR by the IVW method revealed a significant causal estimate for the genetically predicted AF with LTL shortening (OR = 0.989; 95% CI = 0.981–0.997; *p* = 0.007). The results were congruent with the estimations from MR-Egger, Weight Mode, or RAPS (Figure 2A). No outliers were found by MR-PRESSO estimates. The scatter plot contrasted each SNP effect on the AF against its effect on the LTL (Figure 3A) and the causal effect of each single AF-associated SNP on LTL was shown in the forest plot (Supplementary Figure 1A). No significant violation is indicated by the egger intercept test for potential horizontal pleiotropy (*p* > 0.05). In addition, no SNPs altered MR estimates based on the leave-one-out analysis (Supplementary Figure 2A) and the single-SNP analysis showed the distribution of the AF effect on LTL was symmetrical, indicating the stability and reliability of the forward MR results (Supplementary Figure 3A).

Meanwhile, In the eQTL and pQTL analyses, the eQTL-SNPs and pQTL-SNPs related with AF served as the novel exposure dataset. In eQTL-based MR analysis, the IVW method revealed a statistically significant causal effect of genetically predicted AF on LTL shortening (OR = 0.988; 95% CI = 0.980–0.996; *p* = 0.005). The estimates from MR-Egger, Weight Mode, RAPS, or MR-PRESSO were consistent with the results (Figure 2A). And in pQTL-based MR analysis, the causal estimations derived by IVW, Weight Median, Simple Mode, Weight Mode, RAPS, MR-PRESSO, and Maximum likelihood were likewise statistically significant (IVW: OR = 0.975; 95% CI = 0.959–0.992; *p* < 0.005; Figure 2A). The MR-Egger intercept (pleiotropy *p* > 0.05) indicating directional pleiotropy was nonsignificant in eQTL and pQTL analyses. Each SNP effect was shown in the scatter plot (Figures 3B,C) and the forest plot (Supplementary Figures 1B,C). The leave-one-out analysis (Supplementary Figures 2B,C) and single-SNP analysis (Supplementary Figures 3B,C) also indicated that there was no significant disproportionate effect from a particular SNP in the causal estimations.

**Figure 4:**
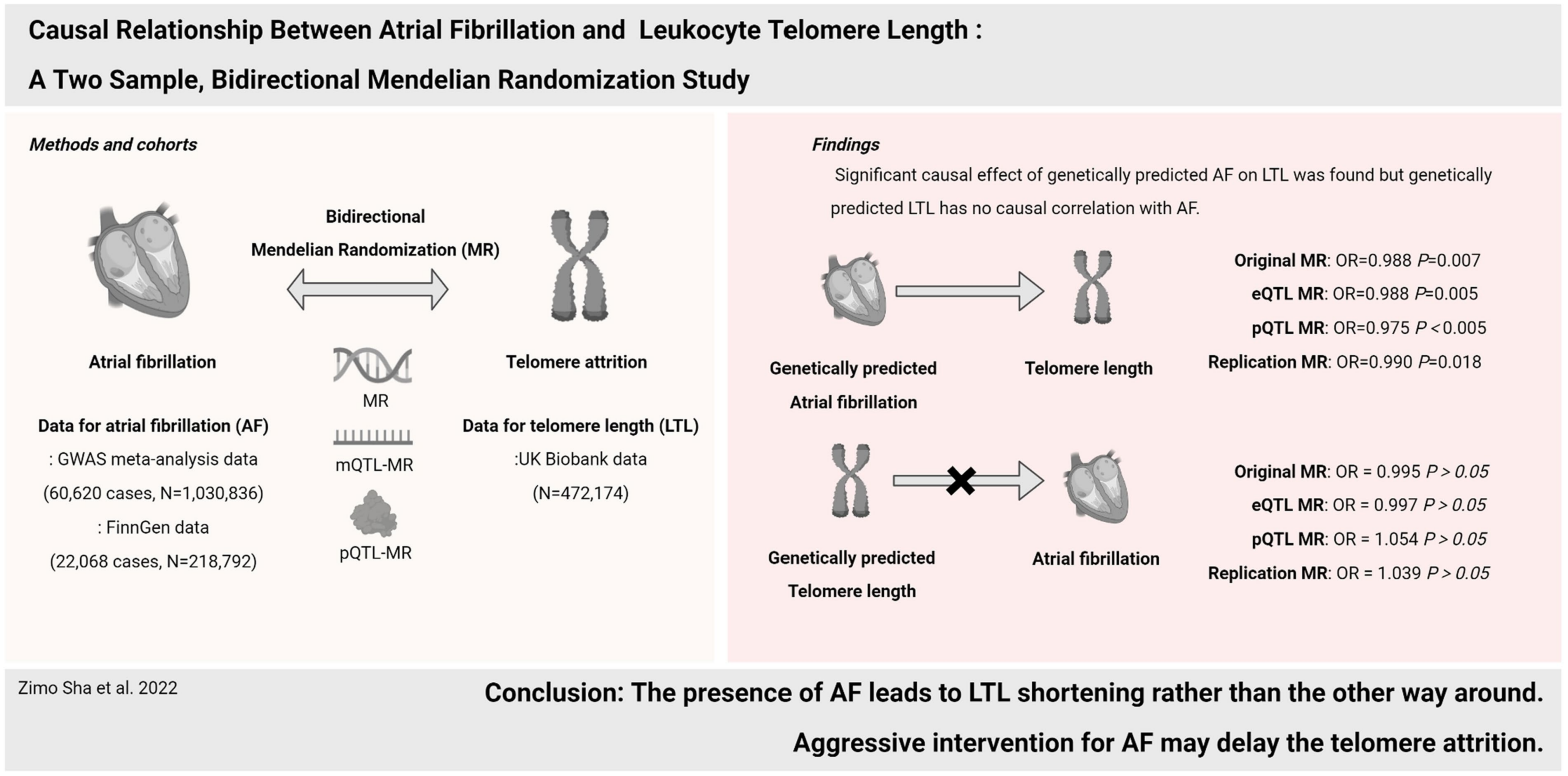
Causal relationship between atrial fibrillation and leukocyte telomere length.

### 3.4. Causal association of LTL with AF *via* reverse MR (original analyses)

In the reverse MR analysis, IVW and other MR methods were consistent indicating that genetically predicted LTL has no significant correlation with AF (IVW: OR = 0.995; 95% CI = 0.914–1.0884; *p* = 0.916; Figure 2B). No outliers were found by MR-PRESSO estimates. The scatter plot compared the SNP effect on the LTL to the SNP effect on AF (Figure 3D) and the forest plot showed causal effect of each single LTL-associated SNP on the risk of AF (Supplementary Figure 1D). Pleiotropy bias was not detected based on egger intercept analysis (*p* > 0.05). The leave-one-out analysis and the single-SNP analysis revealed no SNPs altered MR estimates (Supplementary Figures 2D, 3D).

In eQTL-based MR analysis, IVW did not indicate a causal effect of genetically predicted LTL shortening on AF when the eQTL-SNPs linked with LTL were considered to be novel exposures (OR = 0.999; 95% CI = 0.916–1.091; *p* = 0.995). PQTL-based MR analysis yielded comparable results (OR = 1.055; 95% CI = 0.878–1.267; *p* = 0.570). And the estimates from Weight Median, MR-Egger, Simple Mode, Weight Mode, RAPS, and MR-PRESSO in eQTL-based or pQTL-based MR were consistent with the IVW method (Figure 2B). Each individual SNP effect was represented in the scatter plot (Figures 3E,F). Sensitivity analysis ensures the stability of outcomes (Supplementary Figures 1–3).

### 3.5. Bidirectional causal association of AF with LTL in replication MR analyses

In the replication MR analyses, results were consistent with the original results. IVW revealed causal effect of genetically predicted AF occurrence on LTL shortening (OR = 0.990; 95% CI = 0.982–0.998; *p* = 0.018). However, no causal effect of genetically predicted LTL shortening on the risk of AF was found (OR = 1.100; 95% CI = 0.982–1.232; *p* = 0.099). We presented the detailed results in Supplementary Figure 4.

## 4. Discussion

In the present study, the bi-directional MR approach was utilized to indicate the causal effect of AF on LTL shortening, rather than the other way around. Importantly, the results were additionally validated by eQTL, pQTL-based MR, and several sensitivity analyses. The findings support aggressive intervention for atrial fibrillation as a potential determinant of LTL (Figure 4).

A previous cross-sectional study conducted by Carlquist et al. also examined the association between LTL and AF (8). The study enrolled 3,576 individuals from the Intermountain Heart Collaborative Study. Total of 379 of them were diagnosed with atrial fibrillation, whose average age was 62.9 years. After adjusting for age and cardiovascular risk factors, the study concluded similarly to us that telomere length was shorter in atrial fibrillation patients (*p* = 0.0165). Another case–control study with data from the AFLMU, which is one of the largest cohorts to date with 2,475 AF patients also showed individuals with AF have significantly shorter telomere length compared to controls (*p* < 0.01) (37). And this relation remained significant after multi-variable adjustment for sex, BMI, DM, and hypertension. Unlike these cross-sectional studies, however, community-based prospective cohort studies have reached contradictory conclusions. The Framingham Heart Study which involved 1,143 participants and a 15-year follow-up period, was unable to confirm the causal relationship between telomere shortening and AF (13). The CHS Study, which enrolled 1,675 older individuals with an average age of 72.2 years, also revealed no evidence of an association between LTL and incident AF (38). After 11.4 years of follow-up on 7,775 individuals from the PREVEND cohort, Siland found the incident AF was inversely related to the telomere length but after considering age or AF risk factors, the correlation between telomere length and incident AF was no longer significant (39). These contradiction between cross-sectional and prospective studies could be due to confounding bias or reverse causality. However, Mendelian randomization studies may also have some shortcomings, and the results of the analysis may be affected by the presence of pleiotropy and collider bias, subsequently resulting in some biologically meaningful results that are difficult to interpret. A letter published by Liu et al. also used Mendelian randomization to investigate the relationship between AF and telomere length, and they concluded that AF did not affect telomeres, whereas telomere length was positively associated with AF incidence (40). Although the authors considered the issue of sample overlap, the second basic assumption of Mendelian randomization analysis remained unfulfilled, namely that instrumental variables cannot be associated with confounders between exposure and outcome. The present Mendelian randomization study employs instrumental variables to eliminate confounding factors and offers a new perspective on the reverse causality between telomeres and atrial fibrillation. We used the Phenoscanner database to exclude SNPs associated with confounders and eQTL and pQTL analyses provided more statistically significant results. It is possible be that the presence of AF leads to the shortening of peripheral blood leukocytes telomeres, as opposed to short telomeres directly promoting the occurrence of atrial fibrillation.

Several pathogenic processes linked with AF may explain its effects on telomeres. First, AF causes a decrease in cardiac output of approximately 25%, affecting activity tolerance and frailty indices, which may accelerate systemic telomere shortening (41, 42). Second, the thrombosis and hemodynamic abnormalities induced by AF cause damage to other organs, such as the lungs and kidneys, and the reduction in the function of these organs can also result in telomere attrition (43–45). Third, animal experiments have also shown that atrial fibrillation leads to the accumulation of reactive oxygen species and elevated levels of inflammation, which are also potential factors for DNA and telomere damage (46). Conversely, telomere shortening may also be associated with several risk factors for AF, but with opposite effects. For instance, telomere length is positively associated with coronary heart disease but negatively associated with hypertension and certain cancers (18). The superposition of these effects may lead to an overall lack of causal association between LTL and AF in a large population study.

There are several important strengths in this study. First, the data was derived from the three most recent GWAS datasets containing 472,174 and 1,030,836 individuals of European ancestry in our original MR analyses and 218,792 individuals in replication MR, preventing the simple error in previous cohort studies. Second, LD-independent SNPs with genome-wide association were selected to detect the causal link, and each single SNP was examed in the Phenoscanner database to eschew the confounding factors and pleiotropic effects. Third, eQTL and pQTL in this study refer to which of the instrumental SNPs are associated with transcription and translation when atrial fibrillation or telomere length is seen as exposure, rather than the screening of protein targets in previous studies (47, 48). This makes them, together with other sensitivity analyses, guarantee the stability of the results.

Nonetheless, some limitations in this study must be noted. First, the bidirectional GWAS data were from individuals of European ancestry, thus the results may not be fully representative of the entire population and might not be generalizable. And the exposure and outcome GWAS data may overlap due to the large sample size and single population. To limit the influence of sample overlap, we employed the AF data from the FinnGen cohort and the LTL data from the UKB in the replication analysis, and the results were identical to those of the original analysis. Meanwhile, Maximum likelihood analysis was applied to exclude the effect of bias from sample overlap. Second, AF is a progressive condition that can progress from paroxysmal to persistent and in which telomere length is irreversibly shortened over time, but the relationship between LTL and different AF subtypes cannot be distinguished in this study. Third, GWAS data of TL was from leukocytes rather than atrial tissues, though it is believed that peripheral LTL measures also serve as a marker of TL in other tissues for its strong correlation across tissues (49). Fourth, with the deepening of telomere research, it has been found that in addition to telomere length, telomere structure is also involved in crucial biological functions (50). However, the telomere data in this study were derived from qPCR results and were unable to examine telomere structural changes or telomere damage. Although some eQTL and pQTL-SNP associated with shelterin, which is the protein complex maintaining telomere length and structure were served as IVs in this study, the role of cardiomyocyte telomere structural changes in AF or cardiac function should still be focused on in future research.

In summary, these data shed fresh light on the causal direction between AF and LTL that the presence of AF leads to LTL shortening rather than the reverse. It also suggested telomere lengthening therapy has limited effect on the prevention of new-onset atrial fibrillation, but patients with long-term atrial fibrillation should pay more attention to lifestyle or certain nutritional supplements to prevent telomere shortening, and the intervention for new-onset atrial fibrillation should also be more aggressive to avoid its extensive adverse effects and even aging-promoting effects.

## Data availability statement

The original contributions presented in the study are included in the article/Supplementary material, further inquiries can be directed to the corresponding authors.

## Author contributions

ZS, TH, LW, and YL contributed to the conception and design of the study. ZS and TH performed the main statistical analysis and finished the paper writing. TZ, YB and YD offered guidance and methods for the statistical analysis. LW, YL, JY and QJ offered clinical advice. LW and YL take responsibility for the contents of the article. All authors participated in drafting the manuscript. All authors reviewed the manuscript and approved the final version to be published.

## Funding

This work was supported by the National Natural Science Foundation of China (Nos. 81870250 and 81900290).

## Conflict of interest

The authors declare that the research was conducted in the absence of any commercial or financial relationships that could be construed as a potential conflict of inter.

## Publisher’s note

All claims expressed in this article are solely those of the authors and do not necessarily represent those of their affiliated organizations, or those of the publisher, the editors and the reviewers. Any product that may be evaluated in this article, or claim that may be made by its manufacturer, is not guaranteed or endorsed by the publisher.
