# Supplementary material for: Causal relationship between atrial fibrillation and leukocyte telomere length: A two sample, bidirectional Mendelian randomization study
Source: Front Cardiovasc Med. 2023 Feb 15;10:1093255. doi: 10.3389/fcvm.2023.1093255 (PMC9975167; doi:10.3389/fcvm.2023.1093255)
Supplement: Supplementary file 1 [file Data_Sheet_1.PDF]

SNPs for AF in the forward MR analysis

														Reasons for removing these SNPs		
**chronome**	**position**	**SNP**	**effect_allele**	**other_allele**	**beta**	**se**	**pval**	**sample size**	**EAF**	**exposure**	**ID.exposure**	**eQTL-SNP**	**pQTL-SNP**	**palindromic or incompatible**	**not included in outcome**	**violations of assumption**
14	35184323	rs10141892	C	T	-0.0452	0.0068	2.95E-11	1030836	0.5833	Atrial fibrillation	ebi-a-GCST006414	eQTL	pQTL			
4	148937537	rs10213171	G	C	0.091	0.0134	1.32E-11	1030836	0.0609	Atrial fibrillation	ebi-a-GCST006414	eQTL				
10	77936670	rs10458662	G	T	0.0544	0.0088	6.93E-10	1030836	0.1722	Atrial fibrillation	ebi-a-GCST006414				not included	
5	127819132	rs10520002	A	G	0.0626	0.0113	2.85E-08	1030836	0.0988	Atrial fibrillation	ebi-a-GCST006414	eQTL				
4	174447349	rs10520260	G	A	-0.0457	0.0073	3.36E-10	1030836	0.3214	Atrial fibrillation	ebi-a-GCST006414	eQTL				
1	203026214	rs10753933	G	T	-0.0609	0.0067	9.84E-20	1030836	0.5518	Atrial fibrillation	ebi-a-GCST006414				not included	
12	123327900	rs10773657	A	C	-0.0575	0.0103	2.54E-08	1030836	0.862	Atrial fibrillation	ebi-a-GCST006414					BMI
3	111554426	rs10804493	A	G	0.0558	0.007	1.63E-15	1030836	0.6505	Atrial fibrillation	ebi-a-GCST006414	eQTL				
9	97713459	rs10821415	A	C	0.0821	0.0067	2.92E-34	1030836	0.4132	Atrial fibrillation	ebi-a-GCST006414				not included	
12	24771967	rs10842383	T	C	-0.0988	0.0095	2.88E-25	1030836	0.1478	Atrial fibrillation	ebi-a-GCST006414				not included	
10	103555611	rs11191116	T	C	-0.041	0.007	4.42E-09	1030836	0.348	Atrial fibrillation	ebi-a-GCST006414					Inflammation
1	154862952	rs11264280	T	C	0.1347	0.0071	3.07E-79	1030836	0.333	Atrial fibrillation	ebi-a-GCST006414	eQTL				
10	105342672	rs11598047	G	A	0.1537	0.009	8.95E-66	1030836	0.1621	Atrial fibrillation	ebi-a-GCST006414					Inflammation
7	116191301	rs11773845	A	C	0.1054	0.0067	2.39E-55	1030836	0.5856	Atrial fibrillation	ebi-a-GCST006414				not included	
6	149399100	rs117984853	T	G	0.1228	0.012	1.34E-24	1030836	0.1013	Atrial fibrillation	ebi-a-GCST006414	eQTL				
10	65321147	rs12245149	A	C	-0.047	0.0067	1.66E-12	1030836	0.4739	Atrial fibrillation	ebi-a-GCST006414					Alcohol
12	76237987	rs12426679	T	C	-0.0391	0.0067	4.95E-09	1030836	0.5278	Atrial fibrillation	ebi-a-GCST006414	eQTL				
3	135814009	rs1278493	A	G	-0.0389	0.0068	8.77E-09	1030836	0.5645	Atrial fibrillation	ebi-a-GCST006414	eQTL				
22	26159289	rs133885	A	G	0.0405	0.0068	2.22E-09	1030836	0.4377	Atrial fibrillation	ebi-a-GCST006414	eQTL				
16	2003016	rs140185678	A	G	0.1659	0.0218	2.43E-14	1030836	0.0351	Atrial fibrillation	ebi-a-GCST006414	eQTL				
4	81164723	rs1458038	T	C	0.0434	0.0072	1.74E-09	1030836	0.3087	Atrial fibrillation	ebi-a-GCST006414				not included	
1	51535039	rs146518726	A	G	0.1605	0.0207	8.27E-15	1030836	0.0328	Atrial fibrillation	ebi-a-GCST006414	eQTL				
17	44874453	rs1563304	T	C	0.0644	0.0092	2.56E-12	1030836	0.178	Atrial fibrillation	ebi-a-GCST006414	eQTL				
5	137364795	rs17171711	T	C	0.1086	0.0087	1.95E-35	1030836	0.1775	Atrial fibrillation	ebi-a-GCST006414	eQTL				
12	26345526	rs17380837	T	C	-0.0501	0.0072	4.80E-12	1030836	0.307	Atrial fibrillation	ebi-a-GCST006414	eQTL				
5	114426668	rs1838747	G	A	0.0391	0.0067	4.13E-09	1030836	0.4954	Atrial fibrillation	ebi-a-GCST006414	eQTL				
4	111701798	rs1906615	T	G	0.3658	0.0081	1.00E-200	1030836	0.1991	Atrial fibrillation	ebi-a-GCST006414	eQTL				
6	87821501	rs2031522	G	A	-0.0436	0.0068	1.47E-10	1030836	0.3764	Atrial fibrillation	ebi-a-GCST006414	eQTL	pQTL			
9	139094773	rs2274115	G	A	0.0487	0.0076	1.69E-10	1030836	0.7003	Atrial fibrillation	ebi-a-GCST006414	eQTL	pQTL			
2	179411665	rs2288327	G	A	0.0919	0.0089	7.26E-25	1030836	0.1564	Atrial fibrillation	ebi-a-GCST006414	eQTL	pQTL			
16	73053022	rs2359171	A	T	0.1746	0.0086	4.65E-91	1030836	0.176	Atrial fibrillation	ebi-a-GCST006414					BMI
2	65284231	rs2540949	T	A	-0.0659	0.0068	2.95E-22	1030836	0.3847	Atrial fibrillation	ebi-a-GCST006414					DM
14	64679960	rs2738413	G	A	-0.0778	0.0067	2.55E-31	1030836	0.5049	Atrial fibrillation	ebi-a-GCST006414	eQTL	pQTL			
4	111543323	rs2739197	G	C	0.116	0.0086	3.20E-41	1030836	0.2458	Atrial fibrillation	ebi-a-GCST006414					
15	80994288	rs2759301	A	G	0.039	0.0067	5.04E-09	1030836	0.4542	Atrial fibrillation	ebi-a-GCST006414					BMI
21	36119111	rs2834618	G	T	-0.0944	0.0112	3.41E-17	1030836	0.1056	Atrial fibrillation	ebi-a-GCST006414	eQTL	pQTL			
2	127433465	rs28387148	T	C	0.0741	0.0113	6.25E-11	1030836	0.1051	Atrial fibrillation	ebi-a-GCST006414	eQTL				
1	10790797	rs284277	A	C	-0.0422	0.0069	1.25E-09	1030836	0.6174	Atrial fibrillation	ebi-a-GCST006414	eQTL				
12	57105938	rs2860482	C	A	-0.054	0.0076	1.21E-12	1030836	0.726	Atrial fibrillation	ebi-a-GCST006414	eQTL				
14	23888183	rs28631169	T	C	0.0522	0.0084	5.35E-10	1030836	0.1982	Atrial fibrillation	ebi-a-GCST006414	eQTL				
1	41544279	rs2885697	T	G	-0.0439	0.007	2.88E-10	1030836	0.6482	Atrial fibrillation	ebi-a-GCST006414	eQTL				
6	36647289	rs3176326	A	G	-0.0626	0.0085	1.42E-13	1030836	0.1982	Atrial fibrillation	ebi-a-GCST006414	eQTL	pQTL			
5	113737062	rs337705	G	T	0.0564	0.0068	1.63E-16	1030836	0.3749	Atrial fibrillation	ebi-a-GCST006414	eQTL	pQTL			
3	66454191	rs34080181	A	G	-0.0446	0.0069	1.28E-10	1030836	0.379	Atrial fibrillation	ebi-a-GCST006414	eQTL				
10	105523416	rs34936990	A	G	0.1294	0.0101	2.95E-37	1030836	0.1207	Atrial fibrillation	ebi-a-GCST006414					Inflammation
6	18210109	rs34969716	A	G	0.0702	0.0078	1.60E-19	1030836	0.3051	Atrial fibrillation	ebi-a-GCST006414	eQTL				
2	213266003	rs35544454	T	A	-0.0589	0.0087	1.10E-11	1030836	0.1918	Atrial fibrillation	ebi-a-GCST006414	eQTL				
13	113872712	rs35569628	C	T	-0.0452	0.008	1.38E-08	1030836	0.223	Atrial fibrillation	ebi-a-GCST006414	eQTL	pQTL			
8	11495702	rs35963991	T	G	0.0525	0.0095	2.80E-08	1030836	0.1494	Atrial fibrillation	ebi-a-GCST006414	eQTL	pQTL			
8	21845619	rs3943207	T	G	-0.0638	0.0103	6.92E-10	1030836	0.1167	Atrial fibrillation	ebi-a-GCST006414					Thyrotoxicosis
1	116297758	rs4073778	A	C	0.0486	0.0067	4.96E-13	1030836	0.5639	Atrial fibrillation	ebi-a-GCST006414	eQTL				
17	37868715	rs4252627	T	C	-0.0415	0.0071	5.63E-09	1030836	0.6679	Atrial fibrillation	ebi-a-GCST006414	eQTL	pQTL			
14	32992334	rs4587869	C	G	0.0716	0.0077	1.19E-20	1030836	0.2849	Atrial fibrillation	ebi-a-GCST006414	eQTL	pQTL			
3	12842223	rs4642101	G	T	0.0706	0.0069	2.95E-24	1030836	0.6397	Atrial fibrillation	ebi-a-GCST006414					DM
22	18597502	rs464901	C	T	-0.0508	0.0072	1.53E-12	1030836	0.3353	Atrial fibrillation	ebi-a-GCST006414	eQTL	pQTL			
11	20010291	rs4757877	G	A	-0.0723	0.0078	2.93E-20	1030836	0.7552	Atrial fibrillation	ebi-a-GCST006414	eQTL				
11	121661507	rs4935786	A	T	-0.0463	0.0079	4.85E-09	1030836	0.7327	Atrial fibrillation	ebi-a-GCST006414	eQTL				
6	118565665	rs4946333	G	A	0.0639	0.0066	5.47E-22	1030836	0.4897	Atrial fibrillation	ebi-a-GCST006414					BMI
15	99268850	rs4965430	G	C	-0.0441	0.0069	1.26E-10	1030836	0.6136	Atrial fibrillation	ebi-a-GCST006414		pQTL		not included	
7	14372009	rs55734480	A	G	0.0548	0.0078	2.20E-12	1030836	0.2494	Atrial fibrillation	ebi-a-GCST006414	eQTL	pQTL			
7	128417044	rs55985730	G	T	0.0867	0.0149	5.24E-09	1030836	0.06	Atrial fibrillation	ebi-a-GCST006414	eQTL				
7	92278116	rs56201652	A	G	-0.0531	0.0075	1.74E-12	1030836	0.267	Atrial fibrillation	ebi-a-GCST006414	eQTL				
2	201168758	rs56326533	C	T	0.0685	0.0068	6.28E-24	1030836	0.3919	Atrial fibrillation	ebi-a-GCST006414	eQTL	pQTL			
1	170587340	rs577676	T	C	-0.0923	0.0067	1.62E-43	1030836	0.4383	Atrial fibrillation	ebi-a-GCST006414	eQTL	pQTL			
3	194800853	rs60902112	T	C	0.0445	0.0079	1.72E-08	1030836	0.2262	Atrial fibrillation	ebi-a-GCST006414	eQTL				
3	69417585	rs62254082	C	T	0.0404	0.007	6.34E-09	1030836	0.3865	Atrial fibrillation	ebi-a-GCST006414	eQTL				
5	168383543	rs62377206	A	G	0.0846	0.0147	8.21E-09	1030836	0.0554	Atrial fibrillation	ebi-a-GCST006414	eQTL				
8	124551975	rs62521286	G	A	0.1202	0.0135	4.50E-19	1030836	0.0663	Atrial fibrillation	ebi-a-GCST006414	eQTL				
7	28415827	rs6462079	A	G	0.0466	0.0076	8.79E-10	1030836	0.7208	Atrial fibrillation	ebi-a-GCST006414	eQTL				
2	26159940	rs6546620	C	T	0.0602	0.0086	3.19E-12	1030836	0.7999	Atrial fibrillation	ebi-a-GCST006414	eQTL	pQTL			

12	133150210	rs6560886	C	T	0.051	0.009	1.49E-08	1030836	0.7884	Atrial fibrillation	ebi-a-GCST006414	eQTL			
5	142818123	rs6580277	G	A	0.067	0.0079	1.64E-17	1030836	0.2369	Atrial fibrillation	ebi-a-GCST006414	eQTL			
5	106427609	rs6596717	A	C	-0.0404	0.0068	3.00E-09	1030836	0.6049	Atrial fibrillation	ebi-a-GCST006414				Smoking
1	154802139	rs6665642	T	C	-0.062	0.0112	3.06E-08	1030836	0.1176	Atrial fibrillation	ebi-a-GCST006414	eQTL			
1	154395946	rs6689306	G	A	-0.046	0.0068	1.36E-11	1030836	0.5872	Atrial fibrillation	ebi-a-GCST006414	eQTL	pQTL		
2	70106832	rs6747542	C	T	-0.0554	0.0067	1.10E-16	1030836	0.4642	Atrial fibrillation	ebi-a-GCST006414	eQTL			
3	89489529	rs6771054	C	T	-0.0457	0.0068	2.42E-11	1030836	0.4035	Atrial fibrillation	ebi-a-GCST006414	eQTL			
3	38771925	rs6790396	G	C	0.0627	0.0068	2.40E-20	1030836	0.5959	Atrial fibrillation	ebi-a-GCST006414	eQTL			
2	145760353	rs67969609	G	C	0.0711	0.0126	1.71E-08	1030836	0.071	Atrial fibrillation	ebi-a-GCST006414	eQTL			
4	111765495	rs6838973	T	C	-0.1514	0.0067	1.03E-111	1030836	0.4406	Atrial fibrillation	ebi-a-GCST006414	eQTL	pQTL		
5	172664163	rs6882776	A	G	-0.0711	0.0074	9.64E-22	1030836	0.2835	Atrial fibrillation	ebi-a-GCST006414	eQTL	pQTL		
8	141740868	rs6994744	C	A	0.0405	0.0066	1.09E-09	1030836	0.4954	Atrial fibrillation	ebi-a-GCST006414	eQTL			
12	70013415	rs71454237	A	G	-0.062	0.0084	1.78E-13	1030836	0.209	Atrial fibrillation	ebi-a-GCST006414	eQTL			
15	73667255	rs7172038	G	T	0.112	0.0089	4.78E-36	1030836	0.1597	Atrial fibrillation	ebi-a-GCST006414	eQTL	pQTL		
17	76772288	rs7224711	T	C	-0.0365	0.0066	3.72E-08	1030836	0.5222	Atrial fibrillation	ebi-a-GCST006414				DM
17	1309850	rs7225165	A	G	-0.0655	0.0111	3.20E-09	1030836	0.1133	Atrial fibrillation	ebi-a-GCST006414	eQTL	pQTL		
1	112458893	rs72694603	T	C	-0.0553	0.0072	2.26E-14	1030836	0.3147	Atrial fibrillation	ebi-a-GCST006414	eQTL			
1	170193825	rs72700114	C	G	0.2021	0.013	3.29E-54	1030836	0.0756	Atrial fibrillation	ebi-a-GCST006414	eQTL			
17	12618680	rs72811294	C	G	-0.072	0.0106	9.67E-12	1030836	0.1131	Atrial fibrillation	ebi-a-GCST006414	eQTL			
2	86594487	rs72926475	A	G	-0.0683	0.0102	2.37E-11	1030836	0.1228	Atrial fibrillation	ebi-a-GCST006414	eQTL	pQTL		
6	122398241	rs72966339	T	C	-0.0616	0.0069	7.42E-19	1030836	0.3679	Atrial fibrillation	ebi-a-GCST006414	eQTL	pQTL		
3	24463235	rs73041705	C	T	-0.0443	0.0073	1.55E-09	1030836	0.2985	Atrial fibrillation	ebi-a-GCST006414	eQTL			
6	16415751	rs73366713	A	G	-0.1035	0.0099	1.53E-25	1030836	0.1396	Atrial fibrillation	ebi-a-GCST006414	eQTL			
4	174642789	rs74500426	T	G	-0.0921	0.0127	4.29E-13	1030836	0.0764	Atrial fibrillation	ebi-a-GCST006414	eQTL			
1	170171598	rs74832855	G	A	0.1216	0.018	1.43E-11	1030836	0.0369	Atrial fibrillation	ebi-a-GCST006414	eQTL			
14	73249419	rs74884082	T	C	-0.0493	0.0078	3.48E-10	1030836	0.2495	Atrial fibrillation	ebi-a-GCST006414	eQTL			
7	74110705	rs74910854	G	A	0.09	0.0164	4.31E-08	1030836	0.0693	Atrial fibrillation	ebi-a-GCST006414				Smoking
8	17913970	rs7508	A	G	0.0711	0.0075	1.69E-21	1030836	0.7109	Atrial fibrillation	ebi-a-GCST006414	eQTL	pQTL		
1	22282619	rs7529220	C	T	0.0621	0.0098	1.98E-10	1030836	0.8469	Atrial fibrillation	ebi-a-GCST006414	eQTL			
2	175512820	rs7574892	A	G	0.0552	0.0067	1.98E-16	1030836	0.4847	Atrial fibrillation	ebi-a-GCST006414	eQTL			
11	128764570	rs76097649	A	G	0.1151	0.0124	1.26E-20	1030836	0.0933	Atrial fibrillation	ebi-a-GCST006414	eQTL			
3	179172979	rs7612445	T	G	0.0493	0.0084	4.81E-09	1030836	0.1879	Atrial fibrillation	ebi-a-GCST006414			not included	
16	2265271	rs77316573	T	C	0.0529	0.0089	3.26E-09	1030836	0.1991	Atrial fibrillation	ebi-a-GCST006414	eQTL			
12	70071513	rs775498	G	A	0.0423	0.0074	1.05E-08	1030836	0.2798	Atrial fibrillation	ebi-a-GCST006414	eQTL			
7	150661409	rs7789146	A	G	-0.0584	0.0087	2.12E-11	1030836	0.1787	Atrial fibrillation	ebi-a-GCST006414	eQTL			
10	75420180	rs7915134	T	C	-0.1168	0.0095	1.42E-34	1030836	0.1439	Atrial fibrillation	ebi-a-GCST006414			not included	
1	147255831	rs79187193	A	G	-0.1162	0.0153	3.15E-14	1030836	0.0569	Atrial fibrillation	ebi-a-GCST006414	eQTL			
18	48708548	rs8088085	C	A	-0.0365	0.0067	4.79E-08	1030836	0.4646	Atrial fibrillation	ebi-a-GCST006414	eQTL			
12	114793240	rs883079	T	C	0.0981	0.0074	2.84E-40	1030836	0.7074	Atrial fibrillation	ebi-a-GCST006414			not included	
13	23368943	rs9506925	T	C	0.0449	0.0075	2.72E-09	1030836	0.2669	Atrial fibrillation	ebi-a-GCST006414	eQTL			
18	46474192	rs9953366	C	T	0.049	0.0073	1.82E-11	1030836	0.6631	Atrial fibrillation	ebi-a-GCST006414	eQTL	pQTL		

AF, atrial fibrillation; DM, diabetes mellitus; BMI, body mass index

SNP, single-nucleotide polymorphism; EAF, effect allele frequency; se, standard error; eQTL, expression quantitative trait loci; pQTL, protein quantitative trait loci
