# Supplementary material for: Causal relationship between atrial fibrillation and leukocyte telomere length: A two sample, bidirectional Mendelian randomization study
Source: Front Cardiovasc Med. 2023 Feb 15;10:1093255. doi: 10.3389/fcvm.2023.1093255 (PMC9975167; doi:10.3389/fcvm.2023.1093255)
Supplement: Supplementary file 2 [file Data_Sheet_2.PDF]

SNPs for LTL in the reverse MR analysis

chronome	position	SNP	effect_allele	other_allele	beta	se	pval	sample size	EAF	exposure	ID.exposure	eQTL-SNP	pQTL-SNP	Reasons for removing these SNPs		
														palindromic or incompatible	not included in outcome	violations of assumption
22	51072289	rs1003322	A	C	0.01417	0.0025	1.00E-08	472174	0.2137	telomere length	ieu-b-4879	eQTL				
8	73958718	rs10112752	A	G	-0.0288	0.002	9.50E-46	472174	0.4304	telomere length	ieu-b-4879	eQTL				
8	95530969	rs1023767	A	G	-0.0184	0.0023	5.00E-15	472174	0.2376	telomere length	ieu-b-4879					BMI
11	5247791	rs10768683	G	C	0.04699	0.0028	1.50E-64	472174	0.841	telomere length	ieu-b-4879	eQTL				
12	122944713	rs10773176	G	A	-0.0172	0.0023	5.20E-14	472174	0.7412	telomere length	ieu-b-4879					BMI
12	111833788	rs10774624	A	G	0.01499	0.0021	2.90E-13	472174	0.5328	telomere length	ieu-b-4879					BMI, DM, smoke
4	9920347	rs10805346	C	T	0.01171	0.002	7.00E-09	472174	0.4393	telomere length	ieu-b-4879	eQTL				
11	9629553	rs10840270	G	C	0.01438	0.0021	1.30E-11	472174	0.6557	telomere length	ieu-b-4879	eQTL	pQTL			
12	11757743	rs10845387	A	G	-0.0141	0.0021	1.50E-11	472174	0.3527	telomere length	ieu-b-4879	eQTL				
10	5870267	rs10905255	T	G	-0.0182	0.002	2.60E-19	472174	0.5792	telomere length	ieu-b-4879	eQTL				
19	4368142	rs11085072	T	C	-0.0132	0.0024	2.60E-08	472174	0.2369	telomere length	ieu-b-4879	eQTL	pQTL			
16	88092092	rs11117354	C	T	0.02325	0.0022	3.40E-26	472174	0.6965	telomere length	ieu-b-4879	eQTL	pQTL			
17	29252703	rs111527438	C	T	0.0125	0.0021	3.10E-09	472174	0.3513	telomere length	ieu-b-4879	eQTL				
16	48283993	rs111950327	C	G	0.02383	0.0041	5.90E-09	472174	0.0636	telomere length	ieu-b-4879	eQTL				
11	108304509	rs11212631	C	T	-0.0193	0.0026	4.70E-14	472174	0.1992	telomere length	ieu-b-4879	eQTL	pQTL			
3	197842892	rs112394943	C	T	-0.0199	0.0028	1.60E-12	472174	0.1627	telomere length	ieu-b-4879				not included	
14	23499321	rs113525195	A	C	-0.0124	0.0022	3.10E-08	472174	0.2903	telomere length	ieu-b-4879	eQTL				
15	50366116	rs11412296	T	TA	0.03323	0.0023	1.40E-45	472174	0.7594	telomere length	ieu-b-4879	eQTL	pQTL			
3	128318179	rs11426156	T	TA	-0.0116	0.0021	2.20E-08	472174	0.3995	telomere length	ieu-b-4879				not included	
9	34107505	rs11557154	T	C	-0.0344	0.003	1.10E-30	472174	0.13	telomere length	ieu-b-4879					BMI
1	146741960	rs11579626	C	A	0.02651	0.0036	1.30E-13	472174	0.0849	telomere length	ieu-b-4879	eQTL	pQTL			
1	114419489	rs11584821	T	C	-0.0307	0.0026	3.00E-31	472174	0.1762	telomere length	ieu-b-4879	eQTL				
18	709396	rs116863223	A	G	-0.0818	0.0094	2.60E-18	472174	0.0118	telomere length	ieu-b-4879	eQTL				
20	62157200	rs11699829	A	G	0.0642	0.006	1.50E-26	472174	0.0341	telomere length	ieu-b-4879					BMI
10	103855348	rs117034449	A	G	0.03744	0.0067	2.10E-08	472174	0.0233	telomere length	ieu-b-4879	eQTL				
7	159117178	rs117407747	T	C	0.04505	0.0061	1.80E-13	472174	0.0276	telomere length	ieu-b-4879	eQTL				
20	62574274	rs117512405	A	G	-0.079	0.0082	9.50E-22	472174	0.017	telomere length	ieu-b-4879	eQTL				
7	124779510	rs117630647	A	G	0.05957	0.0072	1.40E-16	472174	0.0213	telomere length	ieu-b-4879	eQTL				
7	50257703	rs11769630	A	T	-0.0257	0.0039	4.30E-11	472174	0.0722	telomere length	ieu-b-4879	eQTL				
8	56664524	rs11991877	A	T	-0.0301	0.0032	3.20E-21	472174	0.8893	telomere length	ieu-b-4879	eQTL				
12	24762109	rs12369950	C	T	-0.0178	0.0029	8.00E-10	472174	0.1407	telomere length	ieu-b-4879	eQTL				
10	101276256	rs12412214	A	G	-0.0245	0.0022	3.40E-28	472174	0.2798	telomere length	ieu-b-4879	eQTL				
17	2247982	rs12451892	C	T	-0.0116	0.0021	2.20E-08	472174	0.3805	telomere length	ieu-b-4879					DM
20	35525640	rs1291143	C	A	0.04931	0.0028	1.80E-69	472174	0.849	telomere length	ieu-b-4879					Alcohol
16	90141355	rs12925933	C	A	-0.0147	0.0021	7.00E-12	472174	0.6622	telomere length	ieu-b-4879	eQTL				
16	9072085	rs12932179	G	A	-0.0136	0.002	1.80E-11	472174	0.5614	telomere length	ieu-b-4879	eQTL				
3	101267385	rs13062095	C	T	0.01386	0.0021	9.70E-11	472174	0.3278	telomere length	ieu-b-4879	eQTL				
22	50971631	rs131797	T	TAAAAA	0.02438	0.0024	6.80E-25	472174	0.2356	telomere length	ieu-b-4879				not included	
7	23930316	rs13230646	C	T	-0.0173	0.0023	8.90E-14	472174	0.2489	telomere length	ieu-b-4879	eQTL	pQTL			
13	41695100	rs1332941	G	A	0.02566	0.0027	5.90E-21	472174	0.8205	telomere length	ieu-b-4879					BMI
14	73418095	rs137901416	A	G	0.04572	0.0033	4.70E-43	472174	0.1003	telomere length	ieu-b-4879	eQTL	pQTL			
18	729871	rs139669835	T	C	-0.0613	0.0105	6.10E-09	472174	0.0094	telomere length	ieu-b-4879	eQTL				
1	92842367	rs139795227	C	A	0.05994	0.0087	6.70E-12	472174	0.014	telomere length	ieu-b-4879					Alcohol
5	78954683	rs141214782	TTATC	T	-0.0247	0.0034	2.00E-13	472174	0.1012	telomere length	ieu-b-4879	eQTL				
20	62488152	rs142426306	T	C	-0.0505	0.0054	8.70E-21	472174	0.0395	telomere length	ieu-b-4879	eQTL				
6	26360443	rs142730696	TTTTTC	T	0.02169	0.003	6.30E-13	472174	0.8641	telomere length	ieu-b-4879				not included	
20	62291767	rs143190905	T	G	-0.0724	0.0037	1.60E-85	472174	0.0804	telomere length	ieu-b-4879	eQTL				
17	76183233	rs144204502	T	C	-0.1006	0.0091	3.40E-28	472174	0.0126	telomere length	ieu-b-4879	eQTL	pQTL			
1	94322469	rs145114957	G	C	0.02726	0.005	4.60E-08	472174	0.0426	telomere length	ieu-b-4879	eQTL	pQTL			
18	708207	rs150150565	T	C	0.06376	0.0074	6.80E-18	472174	0.0215	telomere length	ieu-b-4879	eQTL				
6	29748690	rs1611236	A	G	-0.016	0.0021	6.10E-14	472174	0.3269	telomere length	ieu-b-4879					
18	42070981	rs16978028	T	A	-0.0299	0.0029	8.20E-26	472174	0.1437	telomere length	ieu-b-4879	eQTL				
12	57082058	rs17445108	A	G	-0.0169	0.003	2.00E-08	472174	0.127	telomere length	ieu-b-4879	eQTL				
15	42032383	rs17677991	G	C	0.02227	0.0021	4.40E-26	472174	0.3421	telomere length	ieu-b-4879					DM
2	210673445	rs17803849	T	C	0.02732	0.002	4.20E-41	472174	0.4052	telomere length	ieu-b-4879	eQTL				
16	14652220	rs182059586	C	T	-0.0571	0.0068	4.90E-17	472174	0.0251	telomere length	ieu-b-4879	eQTL				
5	138914024	rs185174247	A	G	0.03728	0.0044	1.10E-17	472174	0.0561	telomere length	ieu-b-4879	eQTL				
2	54473646	rs188918174	T	C	0.04031	0.0054	1.20E-13	472174	0.0361	telomere length	ieu-b-4879	eQTL				
12	88955469	rs1907702	A	G	0.01502	0.0024	5.90E-10	472174	0.7668	telomere length	ieu-b-4879	eQTL				
14	96181360	rs1957937	T	A	0.02094	0.0027	1.90E-14	472174	0.1602	telomere length	ieu-b-4879	eQTL				
7	159119220	rs1985369	G	A	-0.0312	0.003	3.60E-25	472174	0.8682	telomere length	ieu-b-4879	eQTL				
6	29877483	rs201558190	C	T	-0.0182	0.0022	6.40E-17	472174	0.3633	telomere length	ieu-b-4879				not included	
2	54488018	rs202034370	T	TA	0.10278	0.0065	2.60E-56	472174	0.9754	telomere length	ieu-b-4879				not included	
7	99780283	rs2056726	A	G	-0.0228	0.0024	7.90E-21	472174	0.2144	telomere length	ieu-b-4879					Alcohol
3	49936102	rs2230590	C	T	-0.0158	0.002	3.60E-15	472174	0.5109	telomere length	ieu-b-4879					Alcohol
18	51798047	rs2276182	G	C	0.02335	0.002	2.80E-30	472174	0.4032	telomere length	ieu-b-4879	eQTL				
4	2255063	rs2282764	G	A	-0.0224	0.0029	9.30E-15	472174	0.1424	telomere length	ieu-b-4879	eQTL				
11	47440758	rs2293579	A	G	-0.0129	0.0021	3.30E-10	472174	0.3863	telomere length	ieu-b-4879					BMI, DM
8	21846586	rs2306646	C	G	-0.0209	0.002	3.30E-25	472174	0.5595	telomere length	ieu-b-4879			being palindromic		
7	76310784	rs2538745	C	T	-0.0129	0.0021	3.10E-10	472174	0.6028	telomere length	ieu-b-4879				not included	
2	17841243	rs2555104	C	A	-0.014	0.002	6.60E-12	472174	0.4343	telomere length	ieu-b-4879	eQTL				
6	31794592	rs2763979	T	C	-0.0278	0.0021	1.30E-40	472174	0.3597	telomere length	ieu-b-4879					DM
5	1415068	rs28363070	A	G	0.07556	0.0096	3.50E-15	472174	0.0134	telomere length	ieu-b-4879	eQTL				
22	17469049	rs28502153	A	C	-0.0216	0.0021	1.20E-25	472174	0.378	telomere length	ieu-b-4879					BMI
12	123895906	rs28577594	C	G	0.01877	0.0022	5.40E-17	472174	0.7098	telomere length	ieu-b-4879	eQTL				
16	82200103	rs2967355	C	A	-0.0462	0.0024	4.00E-83	472174	0.7743	telomere length	ieu-b-4879	eQTL				
1	768253	rs2977608	C	A	0.01295	0.0023	3.00E-08	472174	0.7439	telomere length	ieu-b-4879	eQTL				
14	20812951	rs3093888	A	G	-0.029	0.0045	1.50E-10	472174	0.0513	telomere length	ieu-b-4879	eQTL				

14	91971787	rs34550383	C	CT	-0.0192	0.002	8.60E-22	472174	0.5474	telomere length	ieu-b-4879				not included	
9	826585	rs34896435	G	C	0.01546	0.0021	6.40E-14	472174	0.4692	telomere length	ieu-b-4879				not included	
3	169486508	rs35446936	A	G	-0.094	0.0023	1.00E-200	472174	0.2437	telomere length	ieu-b-4879	eQTL				
4	122729413	rs35500378	CACTT	C	0.01447	0.0021	2.00E-12	472174	0.6105	telomere length	ieu-b-4879				not included	
20	62321128	rs35640778	A	G	-0.209	0.007	9.59E-195	472174	0.0208	telomere length	ieu-b-4879	eQTL	pQTL			
2	54622978	rs376641875	C	CATAA	-0.0266	0.0042	3.30E-10	472174	0.9289	telomere length	ieu-b-4879				not included	
1	41231032	rs3767952	A	G	0.01345	0.0024	1.80E-08	472174	0.2267	telomere length	ieu-b-4879	eQTL	pQTL			
16	69406986	rs3785074	G	A	0.02386	0.0022	2.60E-27	472174	0.2897	telomere length	ieu-b-4879	eQTL	pQTL			
18	658423	rs3891167	G	A	-0.0426	0.0024	1.20E-70	472174	0.2534	telomere length	ieu-b-4879	eQTL				
1	45252015	rs41269079	A	T	0.01536	0.0025	1.70E-09	472174	0.189	telomere length	ieu-b-4879	eQTL				
20	62375508	rs41304832	A	G	0.06117	0.0093	5.00E-11	472174	0.0124	telomere length	ieu-b-4879	eQTL	pQTL			
19	45411941	rs429358	C	T	0.01735	0.0028	3.80E-10	472174	0.154	telomere length	ieu-b-4879					BMI
1	110910397	rs4498805	T	G	0.01506	0.002	5.70E-14	472174	0.5466	telomere length	ieu-b-4879	eQTL				
16	28413517	rs450962	G	A	0.01428	0.0025	5.90E-09	472174	0.2838	telomere length	ieu-b-4879	eQTL	pQTL			
19	33752994	rs4530278	T	G	0.01388	0.0021	1.50E-11	472174	0.5982	telomere length	ieu-b-4879	eQTL	pQTL			
14	65543102	rs45604339	T	C	-0.0204	0.0021	4.30E-22	472174	0.3424	telomere length	ieu-b-4879	eQTL				
3	160042459	rs4616688	T	G	-0.0173	0.002	4.50E-18	472174	0.5254	telomere length	ieu-b-4879	eQTL				
4	48843372	rs4695407	G	A	0.01415	0.002	1.50E-12	472174	0.5078	telomere length	ieu-b-4879	eQTL				
17	7760397	rs4724	A	G	-0.0547	0.0031	9.80E-69	472174	0.1166	telomere length	ieu-b-4879	eQTL				
7	128678236	rs4731541	G	C	-0.0206	0.0021	1.40E-23	472174	0.6249	telomere length	ieu-b-4879	eQTL				
9	109639970	rs4743037	T	C	0.0148	0.0024	5.10E-10	472174	0.2309	telomere length	ieu-b-4879	eQTL	pQTL			
5	132397351	rs55747751	A	G	-0.0212	0.0038	1.70E-08	472174	0.0774	telomere length	ieu-b-4879	eQTL				
16	70187811	rs56061761	A	G	-0.0204	0.0022	6.90E-20	472174	0.3326	telomere length	ieu-b-4879	eQTL	pQTL			
2	29098543	rs56178008	A	T	0.01437	0.002	9.70E-13	472174	0.4375	telomere length	ieu-b-4879			being palindromic		
17	41456413	rs56799554	G	A	-0.026	0.0027	3.00E-22	472174	0.1702	telomere length	ieu-b-4879	eQTL				
15	74336633	rs5742915	C	T	0.01934	0.002	1.60E-21	472174	0.4458	telomere length	ieu-b-4879					BMI
17	1666218	rs59409453	G	A	0.02021	0.0023	1.60E-18	472174	0.7306	telomere length	ieu-b-4879	eQTL				
22	45790132	rs6007020	C	T	0.01449	0.0021	4.80E-12	472174	0.3678	telomere length	ieu-b-4879	eQTL	pQTL			
20	66370	rs6054257	A	G	-0.0142	0.0025	1.10E-08	472174	0.7935	telomere length	ieu-b-4879	eQTL	pQTL			
11	108177097	rs611646	A	T	-0.0368	0.002	3.50E-73	472174	0.4087	telomere length	ieu-b-4879	eQTL	pQTL			
7	67200	rs61405042	T	C	-0.0502	0.006	8.50E-17	472174	0.0293	telomere length	ieu-b-4879	eQTL				
5	1294166	rs61748181	T	C	-0.0592	0.006	2.80E-23	472174	0.0289	telomere length	ieu-b-4879	eQTL				
4	164028105	rs6536702	A	G	0.05341	0.0024	9.40E-111	472174	0.7746	telomere length	ieu-b-4879	eQTL				
10	105645725	rs6584579	G	A	0.01149	0.002	2.00E-08	472174	0.3989	telomere length	ieu-b-4879	eQTL				
1	151402045	rs6587577	G	A	-0.0182	0.0026	4.80E-12	472174	0.8263	telomere length	ieu-b-4879	eQTL	pQTL			
11	128500215	rs6590343	G	A	0.01217	0.002	1.50E-09	472174	0.5164	telomere length	ieu-b-4879	eQTL				
1	185315067	rs6659669	T	C	-0.0117	0.0021	1.10E-08	472174	0.6051	telomere length	ieu-b-4879	eQTL				
1	32279629	rs6669563	A	G	0.01824	0.002	2.10E-19	472174	0.4378	telomere length	ieu-b-4879	eQTL				
1	20916238	rs66731853	A	G	-0.0178	0.0022	1.50E-16	472174	0.3173	telomere length	ieu-b-4879	eQTL				
13	71236611	rs670180	A	T	-0.0116	0.002	1.20E-08	472174	0.5691	telomere length	ieu-b-4879			being palindromic		
2	43588302	rs6751209	C	T	-0.014	0.0025	1.60E-08	472174	0.2042	telomere length	ieu-b-4879	eQTL				
3	128215821	rs6776756	A	G	-0.0174	0.002	1.10E-17	472174	0.5976	telomere length	ieu-b-4879	eQTL				
3	170263320	rs6790988	G	A	0.01457	0.0023	1.80E-10	472174	0.7419	telomere length	ieu-b-4879	eQTL	pQTL			
5	1670265	rs6881568	A	C	0.01693	0.0021	3.70E-16	472174	0.3626	telomere length	ieu-b-4879	eQTL				
10	96134685	rs7099229	A	G	-0.0153	0.0022	8.40E-12	472174	0.2733	telomere length	ieu-b-4879	eQTL				
15	56775385	rs7164950	G	A	0.01294	0.002	2.30E-10	472174	0.406	telomere length	ieu-b-4879	eQTL				
17	65705530	rs7209057	A	G	0.01182	0.002	5.70E-09	472174	0.561	telomere length	ieu-b-4879	eQTL				
17	76195153	rs7221585	T	C	0.01433	0.0025	6.70E-09	472174	0.224	telomere length	ieu-b-4879	eQTL				
14	21941148	rs73581419	T	C	0.02298	0.0032	1.30E-12	472174	0.1066	telomere length	ieu-b-4879	eQTL				
5	77973	rs73730598	A	G	0.02736	0.0044	4.70E-10	472174	0.0548	telomere length	ieu-b-4879	eQTL				
17	8064779	rs75664430	G	C	-0.0235	0.0023	3.60E-24	472174	0.248	telomere length	ieu-b-4879					Smoking
16	74678063	rs76065543	T	C	0.03428	0.0029	4.20E-32	472174	0.1375	telomere length	ieu-b-4879	eQTL				
16	50188929	rs76219171	A	G	0.03598	0.0043	7.80E-17	472174	0.0584	telomere length	ieu-b-4879	eQTL				
8	48885436	rs762679	A	T	0.03101	0.0029	1.40E-27	472174	0.8565	telomere length	ieu-b-4879	eQTL				
12	120904895	rs76666449	C	T	0.02951	0.0033	8.20E-19	472174	0.1006	telomere length	ieu-b-4879	eQTL				
5	1285974	rs7705526	A	C	0.0776	0.0022	1.00E-200	472174	0.3266	telomere length	ieu-b-4879	eQTL	pQTL			
10	106280527	rs77231040	C	G	0.09893	0.0135	2.00E-13	472174	0.0057	telomere length	ieu-b-4879	eQTL				
6	28674322	rs7772289	T	G	0.01755	0.002	1.70E-18	472174	0.5031	telomere length	ieu-b-4879	eQTL	pQTL			
2	58979879	rs77732866	A	G	0.01779	0.0029	9.20E-10	472174	0.1376	telomere length	ieu-b-4879					BMI
7	124459852	rs7790856	T	C	-0.0437	0.0022	1.80E-87	472174	0.2891	telomere length	ieu-b-4879	eQTL				
3	72891547	rs78491606	C	A	-0.0756	0.0074	1.90E-24	472174	0.0184	telomere length	ieu-b-4879	eQTL	pQTL			
12	54694560	rs79977579	A	C	0.02815	0.0034	2.30E-16	472174	0.0956	telomere length	ieu-b-4879					
16	3650970	rs80116508	A	G	-0.0353	0.0042	2.00E-17	472174	0.0624	telomere length	ieu-b-4879					
6	204031	rs80324517	A	G	0.03965	0.0047	1.80E-17	472174	0.0483	telomere length	ieu-b-4879	eQTL				
19	57370055	rs8102497	A	G	-0.015	0.002	1.40E-13	472174	0.4318	telomere length	ieu-b-4879	eQTL				
19	22215441	rs8105767	G	A	0.03284	0.0022	2.50E-50	472174	0.2947	telomere length	ieu-b-4879	eQTL				
3	24347800	rs869785	C	T	-0.0147	0.0021	4.40E-12	472174	0.6725	telomere length	ieu-b-4879	eQTL				
4	7044380	rs871134	T	C	-0.0183	0.002	1.70E-19	472174	0.569	telomere length	ieu-b-4879	eQTL				
1	226577306	rs932002	T	C	-0.0402	0.0028	7.30E-47	472174	0.1508	telomere length	ieu-b-4879	eQTL				
6	109601554	rs9398196	G	A	-0.0144	0.002	9.50E-13	472174	0.5201	telomere length	ieu-b-4879	eQTL	pQTL			
11	202253	rs939916	A	G	0.02418	0.0022	6.60E-29	472174	0.67	telomere length	ieu-b-4879	eQTL	pQTL			
10	105675946	rs9419958	C	T	-0.081	0.0029	2.60E-167	472174	0.8614	telomere length	ieu-b-4879					BMI
13	73317585	rs9600019	T	C	0.01271	0.0021	2.40E-09	472174	0.3356	telomere length	ieu-b-4879	eQTL				
3	138244400	rs9878436	T	C	-0.0143	0.002	1.20E-12	472174	0.4344	telomere length	ieu-b-4879					Alcohol
16	3613207	rs9940099	T	G	-0.0336	0.0041	3.20E-16	472174	0.0627	telomere length	ieu-b-4879					
18	78008334	rs9955360	A	C	-0.019	0.003	2.20E-10	472174	0.8693	telomere length	ieu-b-4879				not included	

LTL, leukocyte telomere length; DM, diabetes mellitus; BMI, body mass index
SNP, single-nucleotide polymorphism; EAF, effect allele frequency; se, standard error; eQTL, expression quantitative trait loci; pQTL, protein quantitative trait loci
