# Supplementary material for: Causal relationship between atrial fibrillation and leukocyte telomere length: A two sample, bidirectional Mendelian randomization study
Source: Front Cardiovasc Med. 2023 Feb 15;10:1093255. doi: 10.3389/fcvm.2023.1093255 (PMC9975167; doi:10.3389/fcvm.2023.1093255)
Supplement: Supplementary file 3 [file Data_Sheet_3.PDF]

SNPs for AF in the forward MR analysis

												Reasons for removing these SNPs		
**chronome**	**position**	**SNP**	**effect_allele**	**other_allele**	**beta**	**se**	**pval**	**sample size**	**EAF**	**exposure**	**ID.exposure**	**palindromic or incompatible**	**not included in outcome**	**violations of assumption**
7	14385786	rs10234589	T	C	0.0796	0.0142	1.87E-08	218792	0.2645	Atrial fibrillation	finn-b-I9_AF_EXNONE			
12	114797093	rs10507248	T	G	0.1231	0.0134	4.40E-20	218792	0.6944	Atrial fibrillation	finn-b-I9_AF_EXNONE			
17	45128762	rs1056064	C	T	0.0986	0.0155	2.09E-10	218792	0.2023	Atrial fibrillation	finn-b-I9_AF_EXNONE			
1	203026214	rs10753933	G	T	-0.0801	0.0129	5.36E-10	218792	0.6427	Atrial fibrillation	finn-b-I9_AF_EXNONE			
6	117525810	rs11153652	T	C	0.1224	0.0224	4.60E-08	218792	0.0843	Atrial fibrillation	finn-b-I9_AF_EXNONE			
6	16417733	rs112583508	A	G	-0.1336	0.0209	1.61E-10	218792	0.0986	Atrial fibrillation	finn-b-I9_AF_EXNONE			
1	154845534	rs11264278	C	G	0.1602	0.0129	1.77E-35	218792	0.357	Atrial fibrillation	finn-b-I9_AF_EXNONE			
12	26348429	rs113819537	G	C	-0.0997	0.0134	8.41E-14	218792	0.3114	Atrial fibrillation	finn-b-I9_AF_EXNONE			
1	154793247	rs11583758	T	C	0.1873	0.0218	9.36E-18	218792	0.0895	Atrial fibrillation	finn-b-I9_AF_EXNONE			
10	105342672	rs11598047	G	A	0.1416	0.0213	2.86E-11	218792	0.0924	Atrial fibrillation	finn-b-I9_AF_EXNONE			Inflammation
19	50943038	rs117831371	T	C	0.3708	0.0423	1.74E-18	218792	0.0225	Atrial fibrillation	finn-b-I9_AF_EXNONE			
4	86451003	rs12506039	G	A	-0.0774	0.014	3.55E-08	218792	0.2643	Atrial fibrillation	finn-b-I9_AF_EXNONE			
10	105519364	rs12779135	G	A	0.1572	0.0189	9.37E-17	218792	0.1213	Atrial fibrillation	finn-b-I9_AF_EXNONE			Inflammation
15	80676925	rs12908004	G	A	0.1212	0.0154	4.15E-15	218792	0.2	Atrial fibrillation	finn-b-I9_AF_EXNONE			
2	179490478	rs12988307	C	T	0.14	0.0148	3.65E-21	218792	0.2208	Atrial fibrillation	finn-b-I9_AF_EXNONE			
4	111514751	rs13121747	A	G	-0.1709	0.0138	2.52E-35	218792	0.2933	Atrial fibrillation	finn-b-I9_AF_EXNONE			
16	1982336	rs139277460	G	C	0.4172	0.0551	3.62E-14	218792	0.0132	Atrial fibrillation	finn-b-I9_AF_EXNONE			
6	122388851	rs1402538	A	G	-0.0767	0.0131	4.93E-09	218792	0.3337	Atrial fibrillation	finn-b-I9_AF_EXNONE			
10	75447582	rs147790633	C	T	-0.1529	0.0175	2.05E-18	218792	0.1474	Atrial fibrillation	finn-b-I9_AF_EXNONE			
10	20933401	rs148706031	T	C	0.161	0.0276	5.43E-09	218792	0.0547	Atrial fibrillation	finn-b-I9_AF_EXNONE			
10	69340852	rs148717811	C	T	0.085	0.015	1.43E-08	218792	0.2154	Atrial fibrillation	finn-b-I9_AF_EXNONE			
10	80898969	rs1769758	T	G	0.0684	0.0125	4.45E-08	218792	0.4983	Atrial fibrillation	finn-b-I9_AF_EXNONE			Fatal intracranial haemorrhage
4	111665916	rs1906611	C	T	0.5339	0.0177	1.51E-199	218792	0.1464	Atrial fibrillation	finn-b-I9_AF_EXNONE			Inflammation
16	73051620	rs2106261	T	C	0.1856	0.0148	3.87E-36	218792	0.2244	Atrial fibrillation	finn-b-I9_AF_EXNONE			BMI
1	112451447	rs2120436	T	C	-0.0855	0.0141	1.23E-09	218792	0.2634	Atrial fibrillation	finn-b-I9_AF_EXNONE		not included	
2	65284231	rs2540949	T	A	-0.0774	0.013	2.86E-09	218792	0.3409	Atrial fibrillation	finn-b-I9_AF_EXNONE			DM
1	909419	rs28548431	T	C	-0.0882	0.0156	1.49E-08	218792	0.2096	Atrial fibrillation	finn-b-I9_AF_EXNONE			
6	36646849	rs3176323	C	T	-0.0953	0.0137	2.96E-12	218792	0.2933	Atrial fibrillation	finn-b-I9_AF_EXNONE			
7	116186241	rs3807989	G	A	0.1277	0.0125	2.15E-24	218792	0.5691	Atrial fibrillation	finn-b-I9_AF_EXNONE			
8	11802426	rs4240678	T	C	0.0979	0.0177	3.06E-08	218792	0.3478	Atrial fibrillation	finn-b-I9_AF_EXNONE			BMI
5	137443486	rs529526	C	T	0.1251	0.0137	6.53E-20	218792	0.2849	Atrial fibrillation	finn-b-I9_AF_EXNONE			
7	128417044	rs55985730	G	T	0.1515	0.0228	3.22E-11	218792	0.0812	Atrial fibrillation	finn-b-I9_AF_EXNONE		not included	
1	170591310	rs651386	T	A	-0.0925	0.0128	4.15E-13	218792	0.3796	Atrial fibrillation	finn-b-I9_AF_EXNONE	being palindromic		
4	111765495	rs6838973	T	C	-0.1942	0.0123	5.81E-56	218792	0.5022	Atrial fibrillation	finn-b-I9_AF_EXNONE			
1	170193825	rs72700114	C	G	0.1699	0.0282	1.58E-09	218792	0.0522	Atrial fibrillation	finn-b-I9_AF_EXNONE			
5	142751990	rs72801080	C	T	0.1029	0.0157	6.33E-11	218792	0.189	Atrial fibrillation	finn-b-I9_AF_EXNONE		not included	
12	124808840	rs7294442	C	A	0.0782	0.0143	4.97E-08	218792	0.2493	Atrial fibrillation	finn-b-I9_AF_EXNONE			BMI
11	128766366	rs75557443	T	C	0.1596	0.0211	3.55E-14	218792	0.0969	Atrial fibrillation	finn-b-I9_AF_EXNONE			
2	175487267	rs7590328	A	C	0.1008	0.0153	4.10E-11	218792	0.792	Atrial fibrillation	finn-b-I9_AF_EXNONE			
3	89497082	rs7633500	A	G	-0.0698	0.0125	2.18E-08	218792	0.446	Atrial fibrillation	finn-b-I9_AF_EXNONE			Alcohol
1	170115664	rs76411981	A	T	0.2368	0.0329	6.38E-13	218792	0.0361	Atrial fibrillation	finn-b-I9_AF_EXNONE			
1	147259336	rs76816335	T	G	-0.1505	0.0272	2.99E-08	218792	0.0553	Atrial fibrillation	finn-b-I9_AF_EXNONE		not included	
3	12843368	rs9825233	T	C	0.1089	0.0125	3.23E-18	218792	0.5852	Atrial fibrillation	finn-b-I9_AF_EXNONE			BMI

AF, atrial fibrillation; DM, diabetes mellitus; BMI, body mass index
SNP, single-nucleotide polymorphism; EAF, effect allele frequency; se, standard error
