# Supplementary material for: Causal relationship between atrial fibrillation and leukocyte telomere length: A two sample, bidirectional Mendelian randomization study
Source: Front Cardiovasc Med. 2023 Feb 15;10:1093255. doi: 10.3389/fcvm.2023.1093255 (PMC9975167; doi:10.3389/fcvm.2023.1093255)
Supplement: Supplementary file 4 [file Data_Sheet_4.PDF]

**SNPs for LTL in the reverse MR analysis**

| chronome | position | SNP | effect_allele | other_allele | beta | se | pval | sample size | EAF | exposure | ID.exposure | Reasons for removing these SNPs | | |
|---|---|---|---|---|---|---|---|---|---|---|---|---|---|---|
| | | | | | | | | | | | | palindromic or incompatible | not included in outcome | violations of assumption |
| 22 | 51072289 | rs1003322 | A | C | 0.0141734 | 0.00247546 | 1.00E-08 | 472174 | 0.213742 | telomere length | ieu-b-4879 | | | |
| 8 | 73958718 | rs10112752 | A | G | -0.0287522 | 0.00202518 | 9.50E-46 | 472174 | 0.430369 | telomere length | ieu-b-4879 | | | |
| 8 | 95530969 | rs1023767 | A | G | -0.0183732 | 0.00234772 | 5.00E-15 | 472174 | 0.237595 | telomere length | ieu-b-4879 | | | BMI |
| 11 | 5247791 | rs10768683 | G | C | 0.0469922 | 0.00277015 | 1.50E-64 | 472174 | 0.841033 | telomere length | ieu-b-4879 | | | |
| 12 | 122944713 | rs10773176 | G | A | -0.0172009 | 0.00228534 | 5.20E-14 | 472174 | 0.741214 | telomere length | ieu-b-4879 | | | BMI |
| 12 | 111833788 | rs10774624 | A | G | 0.0149944 | 0.00205494 | 2.90E-13 | 472174 | 0.53276 | telomere length | ieu-b-4879 | | | BMI, DM, smoke |
| 4 | 9920347 | rs10805346 | C | T | 0.0117072 | 0.00202147 | 7.00E-09 | 472174 | 0.439339 | telomere length | ieu-b-4879 | | | |
| 11 | 9629553 | rs10840270 | G | C | 0.014383 | 0.00212494 | 1.30E-11 | 472174 | 0.655684 | telomere length | ieu-b-4879 | | | |
| 12 | 11757743 | rs10845387 | A | G | -0.0141214 | 0.00209396 | 1.50E-11 | 472174 | 0.352666 | telomere length | ieu-b-4879 | | | |
| 10 | 5870267 | rs10905255 | T | G | -0.0182493 | 0.00203099 | 2.60E-19 | 472174 | 0.57919 | telomere length | ieu-b-4879 | | | |
| 19 | 4368142 | rs11085072 | T | C | -0.0131806 | 0.00236713 | 2.60E-08 | 472174 | 0.236909 | telomere length | ieu-b-4879 | | | |
| 16 | 88092092 | rs11117354 | C | T | 0.0232506 | 0.00219601 | 3.40E-26 | 472174 | 0.696513 | telomere length | ieu-b-4879 | | | |
| 17 | 29252703 | rs111527438 | C | T | 0.0125 | 0.00211016 | 3.10E-09 | 472174 | 0.351251 | telomere length | ieu-b-4879 | | | |
| 16 | 48283993 | rs111950327 | C | G | 0.0238271 | 0.00409406 | 5.90E-09 | 472174 | 0.063628 | telomere length | ieu-b-4879 | | | |
| 11 | 108304509 | rs11212631 | C | T | -0.0193458 | 0.00256554 | 4.70E-14 | 472174 | 0.199229 | telomere length | ieu-b-4879 | | not included | |
| 3 | 197842892 | rs112394943 | C | T | -0.0198961 | 0.00281641 | 1.60E-12 | 472174 | 0.162741 | telomere length | ieu-b-4879 | | | |
| 14 | 23499321 | rs113525195 | A | C | -0.0124075 | 0.00224132 | 3.10E-08 | 472174 | 0.290254 | telomere length | ieu-b-4879 | | | |
| 15 | 50366116 | rs11412296 | T | TA | 0.0332259 | 0.00234478 | 1.40E-45 | 472174 | 0.759384 | telomere length | ieu-b-4879 | | | |
| 3 | 128318179 | rs11426156 | T | TA | -0.0115882 | 0.0020709 | 2.20E-08 | 472174 | 0.399525 | telomere length | ieu-b-4879 | | not included | |
| 9 | 34107505 | rs11557154 | T | C | -0.0343719 | 0.00298538 | 1.10E-30 | 472174 | 0.13003 | telomere length | ieu-b-4879 | | | BMI |
| 1 | 146741960 | rs11579626 | C | A | 0.0265113 | 0.00357752 | 1.30E-13 | 472174 | 0.084882 | telomere length | ieu-b-4879 | | | |
| 1 | 114419489 | rs11584821 | T | C | -0.0306517 | 0.00263623 | 3.00E-31 | 472174 | 0.176208 | telomere length | ieu-b-4879 | | | |
| 18 | 709396 | rs116863223 | A | G | -0.0817874 | 0.00937157 | 2.60E-18 | 472174 | 0.011763 | telomere length | ieu-b-4879 | | | |
| 20 | 62157200 | rs11699829 | A | G | 0.0641957 | 0.00602028 | 1.50E-26 | 472174 | 0.034146 | telomere length | ieu-b-4879 | | | BMI |
| 10 | 103855348 | rs117034449 | A | G | 0.0374377 | 0.00667898 | 2.10E-08 | 472174 | 0.023304 | telomere length | ieu-b-4879 | | not included | |
| 7 | 159117178 | rs117407747 | T | C | 0.0450533 | 0.00611706 | 1.80E-13 | 472174 | 0.027571 | telomere length | ieu-b-4879 | | | |
| 20 | 62574274 | rs117512405 | A | G | -0.0790134 | 0.00824611 | 9.50E-22 | 472174 | 0.017043 | telomere length | ieu-b-4879 | | | |
| 7 | 124779510 | rs117630647 | A | G | 0.059565 | 0.00720413 | 1.40E-16 | 472174 | 0.021346 | telomere length | ieu-b-4879 | | | |
| 7 | 50257703 | rs11769630 | A | T | -0.0256807 | 0.00389475 | 4.30E-11 | 472174 | 0.072227 | telomere length | ieu-b-4879 | | | |
| 8 | 56664524 | rs11991877 | A | T | -0.030138 | 0.00318686 | 3.20E-21 | 472174 | 0.889309 | telomere length | ieu-b-4879 | | | |
| 12 | 24762109 | rs12369950 | C | T | -0.0178308 | 0.00290205 | 8.00E-10 | 472174 | 0.140675 | telomere length | ieu-b-4879 | | | |
| 10 | 101276256 | rs12412214 | A | G | -0.0245174 | 0.00222685 | 3.40E-28 | 472174 | 0.279765 | telomere length | ieu-b-4879 | | | |
| 17 | 2247982 | rs12451892 | C | T | -0.0116145 | 0.00207578 | 2.20E-08 | 472174 | 0.380511 | telomere length | ieu-b-4879 | | | DM |
| 20 | 35525640 | rs1291143 | C | A | 0.0493145 | 0.0027991 | 1.80E-69 | 472174 | 0.849026 | telomere length | ieu-b-4879 | | | Alcohol |
| 16 | 90141355 | rs12925933 | C | A | -0.0146622 | 0.00213796 | 7.00E-12 | 472174 | 0.66221 | telomere length | ieu-b-4879 | | | |
| 16 | 9072085 | rs12932179 | G | A | -0.0136257 | 0.0020276 | 1.80E-11 | 472174 | 0.561399 | telomere length | ieu-b-4879 | | | |
| 3 | 101267385 | rs13062095 | C | T | 0.0138552 | 0.00214113 | 9.70E-11 | 472174 | 0.327843 | telomere length | ieu-b-4879 | | | |
| 22 | 50971631 | rs131797 | T | TAAAAA | 0.0243752 | 0.00236561 | 6.80E-25 | 472174 | 0.235625 | telomere length | ieu-b-4879 | | not included | |
| 7 | 23930316 | rs13230646 | C | T | -0.0173277 | 0.00232377 | 8.90E-14 | 472174 | 0.248945 | telomere length | ieu-b-4879 | | | |
| 13 | 41695100 | rs1332941 | G | A | 0.0256552 | 0.00273159 | 5.90E-21 | 472174 | 0.820466 | telomere length | ieu-b-4879 | | | BMI |
| 14 | 73418095 | rs137901416 | A | G | 0.04572 | 0.00332355 | 4.70E-43 | 472174 | 0.100311 | telomere length | ieu-b-4879 | | | |
| 18 | 729871 | rs139669835 | T | C | -0.0612563 | 0.0105346 | 6.10E-09 | 472174 | 0.009365 | telomere length | ieu-b-4879 | | | |
| 1 | 92842367 | rs139795227 | C | A | 0.0599379 | 0.00873247 | 6.70E-12 | 472174 | 0.014021 | telomere length | ieu-b-4879 | | | Alcohol |
| 5 | 78954683 | rs141214782 | TTATC | T | -0.0246687 | 0.00335723 | 2.00E-13 | 472174 | 0.101206 | telomere length | ieu-b-4879 | | | |
| 20 | 62488152 | rs142426306 | T | C | -0.0504903 | 0.00539933 | 8.70E-21 | 472174 | 0.039544 | telomere length | ieu-b-4879 | | | |
| 6 | 26360443 | rs142730696 | TTTTTC | T | 0.0216931 | 0.00301569 | 6.30E-13 | 472174 | 0.864083 | telomere length | ieu-b-4879 | | not included | |
| 20 | 62291767 | rs143190905 | T | G | -0.0723995 | 0.00369421 | 1.60E-85 | 472174 | 0.080404 | telomere length | ieu-b-4879 | | | |
| 17 | 76183233 | rs144204502 | T | C | -0.100574 | 0.00913369 | 3.40E-28 | 472174 | 0.012562 | telomere length | ieu-b-4879 | | | |
| 1 | 94322469 | rs145114957 | G | C | 0.0272605 | 0.00498872 | 4.60E-08 | 472174 | 0.042648 | telomere length | ieu-b-4879 | | | |
| 18 | 708207 | rs150150565 | T | C | 0.063762 | 0.00739877 | 6.80E-18 | 472174 | 0.021455 | telomere length | ieu-b-4879 | | | |
| 6 | 29748690 | rs1611236 | A | G | -0.0160135 | 0.00213359 | 6.10E-14 | 472174 | 0.32687 | telomere length | ieu-b-4879 | | | |
| 18 | 42070981 | rs16978028 | T | A | -0.029945 | 0.00285068 | 8.20E-26 | 472174 | 0.143727 | telomere length | ieu-b-4879 | | | |
| 12 | 57082058 | rs17445108 | A | G | -0.0168922 | 0.00300983 | 2.00E-08 | 472174 | 0.12695 | telomere length | ieu-b-4879 | | | |
| 15 | 42032383 | rs17677991 | G | C | 0.0222664 | 0.00210806 | 4.40E-26 | 472174 | 0.342123 | telomere length | ieu-b-4879 | | | DM |
| 2 | 210673445 | rs17803849 | T | C | 0.0273203 | 0.00203482 | 4.20E-41 | 472174 | 0.405161 | telomere length | ieu-b-4879 | | not included | |
| 16 | 14652220 | rs182059586 | C | T | -0.0571159 | 0.00680853 | 4.90E-17 | 472174 | 0.02511 | telomere length | ieu-b-4879 | | | |
| 5 | 138914024 | rs185174247 | A | G | 0.0372806 | 0.00435145 | 1.10E-17 | 472174 | 0.05609 | telomere length | ieu-b-4879 | | | |
| 2 | 54473646 | rs188918174 | T | C | 0.0403062 | 0.00543604 | 1.20E-13 | 472174 | 0.036106 | telomere length | ieu-b-4879 | | | |
| 12 | 88955469 | rs1907702 | A | G | 0.0150247 | 0.00242651 | 5.90E-10 | 472174 | 0.766771 | telomere length | ieu-b-4879 | | | |
| 14 | 96181360 | rs1957937 | T | A | 0.0209365 | 0.00273361 | 1.90E-14 | 472174 | 0.16018 | telomere length | ieu-b-4879 | | | |
| 7 | 159119220 | rs1985369 | G | A | -0.0311893 | 0.00300952 | 3.60E-25 | 472174 | 0.868178 | telomere length | ieu-b-4879 | | | |
| 6 | 29877483 | rs201558190 | C | T | -0.0181748 | 0.00217477 | 6.40E-17 | 472174 | 0.363271 | telomere length | ieu-b-4879 | | not included | |
| 2 | 54488018 | rs202034370 | T | TA | 0.102784 | 0.00650108 | 2.60E-56 | 472174 | 0.97541 | telomere length | ieu-b-4879 | | not included | |
| 7 | 99780283 | rs2056726 | A | G | -0.0228078 | 0.00243638 | 7.90E-21 | 472174 | 0.214376 | telomere length | ieu-b-4879 | | | Alcohol |
| 3 | 49936102 | rs2230590 | C | T | -0.0158022 | 0.00200806 | 3.60E-15 | 472174 | 0.510897 | telomere length | ieu-b-4879 | | | Alcohol |
| 18 | 51798047 | rs2276182 | G | C | 0.0233529 | 0.00204247 | 2.80E-30 | 472174 | 0.403227 | telomere length | ieu-b-4879 | being palindromic | | |
| 4 | 2255063 | rs2282764 | G | A | -0.0224234 | 0.00289392 | 9.30E-15 | 472174 | 0.142384 | telomere length | ieu-b-4879 | | | |
| 11 | 47440758 | rs2293579 | A | G | -0.012915 | 0.00205481 | 3.30E-10 | 472174 | 0.386274 | telomere length | ieu-b-4879 | | | BMI, DM |
| 8 | 21846586 | rs2306646 | C | G | -0.0209417 | 0.00201898 | 3.30E-25 | 472174 | 0.559475 | telomere length | ieu-b-4879 | being palindromic | | |
| 7 | 76310784 | rs2538745 | C | T | -0.012942 | 0.002056 | 3.10E-10 | 472174 | 0.602841 | telomere length | ieu-b-4879 | | | |
| 2 | 17841243 | rs2555104 | C | A | -0.0139717 | 0.00203498 | 6.60E-12 | 472174 | 0.434255 | telomere length | ieu-b-4879 | | | |
| 6 | 31794592 | rs2763979 | T | C | -0.0277713 | 0.00208092 | 1.30E-40 | 472174 | 0.359721 | telomere length | ieu-b-4879 | | | DM |
| 5 | 1415068 | rs28363070 | A | G | 0.0755557 | 0.00959987 | 3.50E-15 | 472174 | 0.013389 | telomere length | ieu-b-4879 | | | |
| 22 | 17469049 | rs28502153 | A | C | -0.0215916 | 0.00206208 | 1.20E-25 | 472174 | 0.377958 | telomere length | ieu-b-4879 | | | BMI |
| 12 | 123895906 | rs28577594 | C | G | 0.0187657 | 0.00224024 | 5.40E-17 | 472174 | 0.709829 | telomere length | ieu-b-4879 | | | |
| 16 | 82200103 | rs2967355 | C | A | -0.0461595 | 0.00238972 | 4.00E-83 | 472174 | 0.774276 | telomere length | ieu-b-4879 | | | |
| 1 | 768253 | rs2977608 | C | A | 0.0129483 | 0.00233716 | 3.00E-08 | 472174 | 0.743949 | telomere length | ieu-b-4879 | | | |
| 14 | 20812951 | rs3093888 | A | G | -0.028973 | 0.00452459 | 1.50E-10 | 472174 | 0.05131 | telomere length | ieu-b-4879 | | | |

| | | | | | | | | | | | | | | |
|---|---|---|---|---|---|---|---|---|---|---|---|---|---|---|
| 14 | 91971787 | rs34550383 | C | CT | -0.0192236 | 0.00200412 | 8.60E-22 | 472174 | 0.5474 | telomere length | ieu-b-4879 | | not included | |
| 9 | 826585 | rs34896435 | G | C | 0.0154567 | 0.00206109 | 6.40E-14 | 472174 | 0.469158 | telomere length | ieu-b-4879 | | not included | |
| 3 | 169486508 | rs35446936 | A | G | -0.0940025 | 0.00232918 | 1.00E-200 | 472174 | 0.243674 | telomere length | ieu-b-4879 | | | |
| 4 | 122729413 | rs35500378 | CACTT | C | 0.014467 | 0.00205607 | 2.00E-12 | 472174 | 0.610542 | telomere length | ieu-b-4879 | | | |
| 20 | 62321128 | rs35640778 | A | G | -0.209011 | 0.00702087 | 9.59E-195 | 472174 | 0.020757 | telomere length | ieu-b-4879 | | not included | |
| 2 | 54622978 | rs376641875 | C | CATAA | -0.0265863 | 0.00422973 | 3.30E-10 | 472174 | 0.928873 | telomere length | ieu-b-4879 | | not included | |
| 1 | 41231032 | rs3767952 | A | G | 0.0134472 | 0.00238826 | 1.80E-08 | 472174 | 0.226709 | telomere length | ieu-b-4879 | | | |
| 16 | 69406986 | rs3785074 | G | A | 0.023863 | 0.00220455 | 2.60E-27 | 472174 | 0.289672 | telomere length | ieu-b-4879 | | | |
| 18 | 658423 | rs3891167 | G | A | -0.0425685 | 0.00239551 | 1.20E-70 | 472174 | 0.253435 | telomere length | ieu-b-4879 | | | |
| 1 | 45252015 | rs41269079 | A | T | 0.0153617 | 0.0025499 | 1.70E-09 | 472174 | 0.188991 | telomere length | ieu-b-4879 | | | |
| 20 | 62375508 | rs41304832 | A | G | 0.0611702 | 0.0093095 | 5.00E-11 | 472174 | 0.012378 | telomere length | ieu-b-4879 | | | |
| 19 | 45411941 | rs429358 | C | T | 0.0173498 | 0.00277091 | 3.80E-10 | 472174 | 0.153969 | telomere length | ieu-b-4879 | | | BMI |
| 1 | 110910397 | rs4498805 | T | G | 0.0150601 | 0.00200376 | 5.70E-14 | 472174 | 0.546632 | telomere length | ieu-b-4879 | | | |
| 16 | 28413517 | rs450962 | G | A | 0.0142833 | 0.00245428 | 5.90E-09 | 472174 | 0.283779 | telomere length | ieu-b-4879 | | not included | |
| 19 | 33752994 | rs4530278 | T | G | 0.0138793 | 0.0020567 | 1.50E-11 | 472174 | 0.59815 | telomere length | ieu-b-4879 | | | |
| 14 | 65543102 | rs45604339 | T | C | -0.020433 | 0.00211433 | 4.30E-22 | 472174 | 0.34242 | telomere length | ieu-b-4879 | | | |
| 3 | 160042459 | rs4616688 | T | G | -0.0173476 | 0.00200198 | 4.50E-18 | 472174 | 0.525394 | telomere length | ieu-b-4879 | | | |
| 4 | 48843372 | rs4695407 | G | A | 0.0141511 | 0.00199925 | 1.50E-12 | 472174 | 0.507843 | telomere length | ieu-b-4879 | | | |
| 17 | 7760397 | rs4724 | A | G | -0.0547446 | 0.00312441 | 9.80E-69 | 472174 | 0.116598 | telomere length | ieu-b-4879 | | | |
| 7 | 128678236 | rs4731541 | G | C | -0.0206119 | 0.00205962 | 1.40E-23 | 472174 | 0.624901 | telomere length | ieu-b-4879 | | | |
| 9 | 109639970 | rs4743037 | T | C | 0.0147971 | 0.00238094 | 5.10E-10 | 472174 | 0.230874 | telomere length | ieu-b-4879 | | | |
| 5 | 132397351 | rs55747751 | A | G | -0.0211612 | 0.00375164 | 1.70E-08 | 472174 | 0.077375 | telomere length | ieu-b-4879 | | | |
| 16 | 70187811 | rs56061761 | A | G | -0.0203556 | 0.0022296 | 6.90E-20 | 472174 | 0.332642 | telomere length | ieu-b-4879 | | not included | |
| 2 | 29098543 | rs56178008 | A | T | 0.0143739 | 0.00201464 | 9.70E-13 | 472174 | 0.437497 | telomere length | ieu-b-4879 | being palindromic | | |
| 17 | 41456413 | rs56799554 | G | A | -0.0259793 | 0.00267858 | 3.00E-22 | 472174 | 0.170183 | telomere length | ieu-b-4879 | | | |
| 15 | 74336633 | rs5742915 | C | T | 0.0193377 | 0.00202886 | 1.60E-21 | 472174 | 0.445829 | telomere length | ieu-b-4879 | | | BMI |
| 17 | 1666218 | rs59409453 | G | A | 0.0202133 | 0.00230175 | 1.60E-18 | 472174 | 0.730602 | telomere length | ieu-b-4879 | | | |
| 22 | 45790132 | rs6007020 | C | T | 0.0144904 | 0.00209637 | 4.80E-12 | 472174 | 0.367823 | telomere length | ieu-b-4879 | | | |
| 20 | 66370 | rs6054257 | A | G | -0.0141684 | 0.00247729 | 1.10E-08 | 472174 | 0.793522 | telomere length | ieu-b-4879 | | | |
| 11 | 108177097 | rs611646 | A | T | -0.0368309 | 0.00203547 | 3.50E-73 | 472174 | 0.408685 | telomere length | ieu-b-4879 | | | |
| 7 | 67200 | rs61405042 | T | C | -0.0501874 | 0.00602955 | 8.50E-17 | 472174 | 0.029288 | telomere length | ieu-b-4879 | | | |
| 5 | 1294166 | rs61748181 | T | C | -0.059181 | 0.00595394 | 2.80E-23 | 472174 | 0.028928 | telomere length | ieu-b-4879 | | | |
| 4 | 164028105 | rs6536702 | A | G | 0.0534148 | 0.00238875 | 9.40E-111 | 472174 | 0.774647 | telomere length | ieu-b-4879 | | | |
| 10 | 105645725 | rs6584579 | G | A | 0.0114923 | 0.00204674 | 2.00E-08 | 472174 | 0.398876 | telomere length | ieu-b-4879 | | | |
| 1 | 151402045 | rs6587577 | G | A | -0.0182148 | 0.0026359 | 4.80E-12 | 472174 | 0.826346 | telomere length | ieu-b-4879 | | | |
| 11 | 128500215 | rs6590343 | G | A | 0.0121739 | 0.00201444 | 1.50E-09 | 472174 | 0.516385 | telomere length | ieu-b-4879 | | not included | |
| 1 | 185315067 | rs6659669 | T | C | -0.0117091 | 0.00205167 | 1.10E-08 | 472174 | 0.605095 | telomere length | ieu-b-4879 | | | |
| 1 | 32279629 | rs6669563 | A | G | 0.0182358 | 0.00202476 | 2.10E-19 | 472174 | 0.437768 | telomere length | ieu-b-4879 | | | |
| 1 | 20916238 | rs66731853 | A | G | -0.0177791 | 0.00215421 | 1.50E-16 | 472174 | 0.317304 | telomere length | ieu-b-4879 | | | |
| 13 | 71236611 | rs670180 | A | T | -0.0115801 | 0.00203062 | 1.20E-08 | 472174 | 0.569104 | telomere length | ieu-b-4879 | being palindromic | | |
| 2 | 43588302 | rs6751209 | C | T | -0.0140465 | 0.00248465 | 1.60E-08 | 472174 | 0.204231 | telomere length | ieu-b-4879 | | | |
| 3 | 128215821 | rs6776756 | A | G | -0.0174439 | 0.00203747 | 1.10E-17 | 472174 | 0.597562 | telomere length | ieu-b-4879 | | | |
| 3 | 170263320 | rs6790988 | G | A | 0.0145728 | 0.00228428 | 1.80E-10 | 472174 | 0.741902 | telomere length | ieu-b-4879 | | | |
| 5 | 1670265 | rs6881568 | A | C | 0.0169256 | 0.00207735 | 3.70E-16 | 472174 | 0.36258 | telomere length | ieu-b-4879 | | | |
| 10 | 96134685 | rs7099229 | A | G | -0.0153288 | 0.00224403 | 8.40E-12 | 472174 | 0.273301 | telomere length | ieu-b-4879 | | | |
| 15 | 56775385 | rs7164950 | G | A | 0.0129362 | 0.00204001 | 2.30E-10 | 472174 | 0.405979 | telomere length | ieu-b-4879 | | | |
| 17 | 65705530 | rs7209057 | A | G | 0.011819 | 0.00202865 | 5.70E-09 | 472174 | 0.561044 | telomere length | ieu-b-4879 | | | |
| 17 | 76195153 | rs7221585 | T | C | 0.0143271 | 0.00247042 | 6.70E-09 | 472174 | 0.22401 | telomere length | ieu-b-4879 | | | |
| 14 | 21941148 | rs73581419 | T | C | 0.0229838 | 0.00324156 | 1.30E-12 | 472174 | 0.106605 | telomere length | ieu-b-4879 | | | |
| 5 | 77973 | rs73730598 | A | G | 0.0273632 | 0.00439272 | 4.70E-10 | 472174 | 0.054793 | telomere length | ieu-b-4879 | | | |
| 17 | 8064779 | rs75664430 | G | C | -0.0235179 | 0.0023186 | 3.60E-24 | 472174 | 0.248028 | telomere length | ieu-b-4879 | | | Smoking |
| 16 | 74678063 | rs76065543 | T | C | 0.0342843 | 0.00290708 | 4.20E-32 | 472174 | 0.137527 | telomere length | ieu-b-4879 | | | |
| 16 | 50188929 | rs76219171 | A | G | 0.0359839 | 0.00431741 | 7.80E-17 | 472174 | 0.058433 | telomere length | ieu-b-4879 | | | |
| 8 | 48885436 | rs762679 | A | T | 0.0310104 | 0.00285024 | 1.40E-27 | 472174 | 0.856501 | telomere length | ieu-b-4879 | | | |
| 12 | 120904895 | rs76666449 | C | T | 0.0295125 | 0.00333186 | 8.20E-19 | 472174 | 0.100625 | telomere length | ieu-b-4879 | | | |
| 5 | 1285974 | rs7705526 | A | C | 0.0776022 | 0.00216124 | 1.00E-200 | 472174 | 0.326578 | telomere length | ieu-b-4879 | | | |
| 10 | 106280527 | rs77231040 | C | G | 0.0989303 | 0.0134649 | 2.00E-13 | 472174 | 0.005742 | telomere length | ieu-b-4879 | | | |
| 6 | 28674322 | rs7772289 | T | G | 0.017549 | 0.00200003 | 1.70E-18 | 472174 | 0.503081 | telomere length | ieu-b-4879 | | | |
| 2 | 58979879 | rs77732866 | A | G | 0.0177942 | 0.00290595 | 9.20E-10 | 472174 | 0.137597 | telomere length | ieu-b-4879 | | | BMI |
| 7 | 124459852 | rs7790856 | T | C | -0.0437199 | 0.00220526 | 1.80E-87 | 472174 | 0.289139 | telomere length | ieu-b-4879 | | | |
| 3 | 72891547 | rs78491606 | C | A | -0.0756311 | 0.00741168 | 1.90E-24 | 472174 | 0.018433 | telomere length | ieu-b-4879 | | | |
| 12 | 54694560 | rs79977579 | A | C | 0.0281517 | 0.00343182 | 2.30E-16 | 472174 | 0.09555 | telomere length | ieu-b-4879 | | | |
| 16 | 3650970 | rs80116508 | A | G | -0.0352672 | 0.00415151 | 2.00E-17 | 472174 | 0.062356 | telomere length | ieu-b-4879 | | | |
| 6 | 204031 | rs80324517 | A | G | 0.0396515 | 0.00466286 | 1.80E-17 | 472174 | 0.048259 | telomere length | ieu-b-4879 | | | |
| 19 | 57370055 | rs8102497 | A | G | -0.0149654 | 0.0020233 | 1.40E-13 | 472174 | 0.431828 | telomere length | ieu-b-4879 | | | |
| 19 | 22215441 | rs8105767 | G | A | 0.0328384 | 0.00220117 | 2.50E-50 | 472174 | 0.29467 | telomere length | ieu-b-4879 | | | |
| 3 | 24347800 | rs869785 | C | T | -0.0147303 | 0.00212801 | 4.40E-12 | 472174 | 0.672471 | telomere length | ieu-b-4879 | | | |
| 4 | 7044380 | rs871134 | T | C | -0.0182986 | 0.0020263 | 1.70E-19 | 472174 | 0.569032 | telomere length | ieu-b-4879 | | | |
| 1 | 226577306 | rs932002 | T | C | -0.0402052 | 0.00279667 | 7.30E-47 | 472174 | 0.150843 | telomere length | ieu-b-4879 | | | |
| 6 | 109601554 | rs9398196 | G | A | -0.0143586 | 0.00201175 | 9.50E-13 | 472174 | 0.52005 | telomere length | ieu-b-4879 | | | |
| 11 | 202253 | rs939916 | A | G | 0.0241795 | 0.00216724 | 6.60E-29 | 472174 | 0.669967 | telomere length | ieu-b-4879 | | | |
| 10 | 105675946 | rs9419958 | C | T | -0.0810098 | 0.00293847 | 2.60E-167 | 472174 | 0.86139 | telomere length | ieu-b-4879 | | | BMI |
| 13 | 73317585 | rs9600019 | T | C | 0.0127134 | 0.00213096 | 2.40E-09 | 472174 | 0.335579 | telomere length | ieu-b-4879 | | | |
| 3 | 138244400 | rs9878436 | T | C | -0.0143407 | 0.00201819 | 1.20E-12 | 472174 | 0.434393 | telomere length | ieu-b-4879 | | | Alcohol |
| 16 | 3613207 | rs9940099 | T | G | -0.033609 | 0.00411613 | 3.20E-16 | 472174 | 0.062718 | telomere length | ieu-b-4879 | | | |
| 18 | 78008334 | rs9955360 | A | C | -0.0190311 | 0.00299791 | 2.20E-10 | 472174 | 0.869288 | telomere length | ieu-b-4879 | | | |

LTL, leukocyte telomere length; DM, diabetes mellitus; BMI, body mass index
SNP, single-nucleotide polymorphism; EAF, effect allele frequency; se, standard error; eQTL, expression quantitative trait loci; pQTL, protein quantitative trait loci
