# Supplementary material for: Causal relationship between atrial fibrillation and leukocyte telomere length: A two sample, bidirectional Mendelian randomization study
Source: Front Cardiovasc Med. 2023 Feb 15;10:1093255. doi: 10.3389/fcvm.2023.1093255 (PMC9975167; doi:10.3389/fcvm.2023.1093255)
Supplement: Supplementary file 5 [file Data_Sheet_5.PDF]

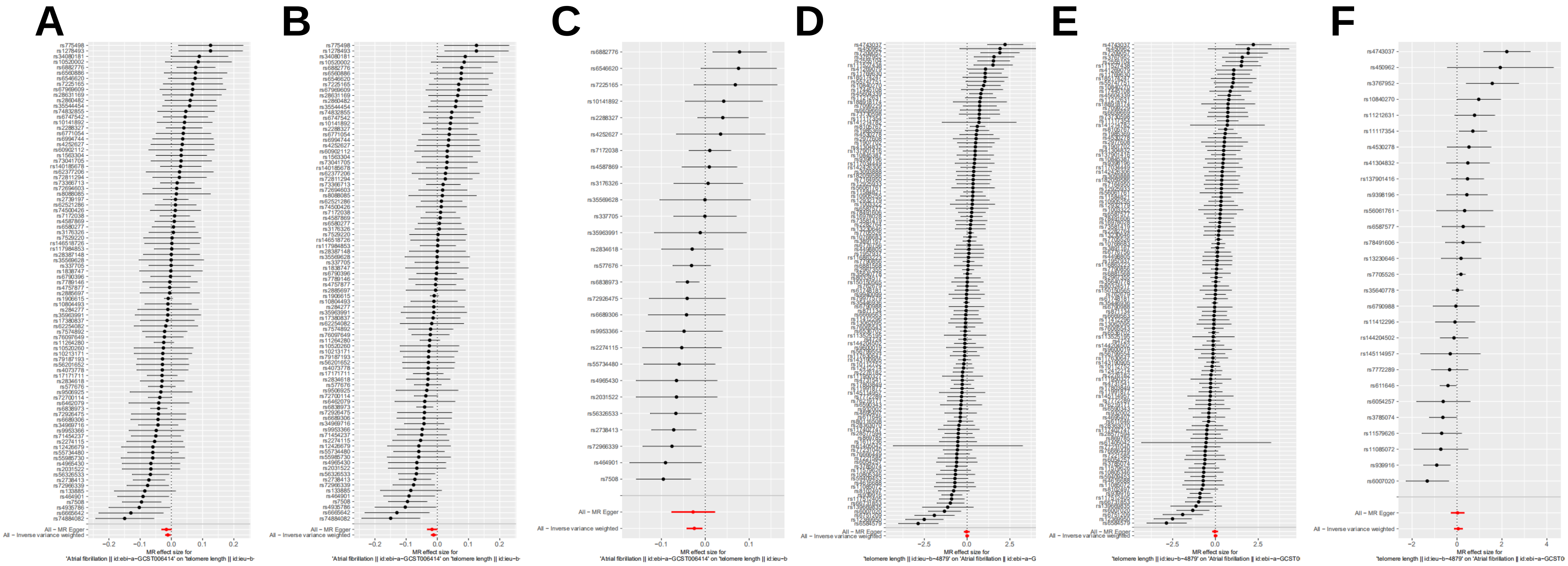

Supplementary Figure 1. Forest plot for mendelian randomization of the association between leukocyte telomere length and atrial fibrillation. A AF-LTL in MR analysis. B AF-LTL in eQTL-MR analysis. C AF-LTL in pQTL-MR analysis. D LTL-AF in MR analysis. E LTL-AF in eQTL-MR analysis. F LTL-AF in pQTL-MR analysis. The dots represent the odds ratio of the corresponding single SNP on the outcome and the lines represent the corresponding 95% confidence interval.
