# Supplementary material for: Causal relationship between atrial fibrillation and leukocyte telomere length: A two sample, bidirectional Mendelian randomization study
Source: Front Cardiovasc Med. 2023 Feb 15;10:1093255. doi: 10.3389/fcvm.2023.1093255 (PMC9975167; doi:10.3389/fcvm.2023.1093255)
Supplement: Supplementary file 7 [file Data_Sheet_7.PDF]

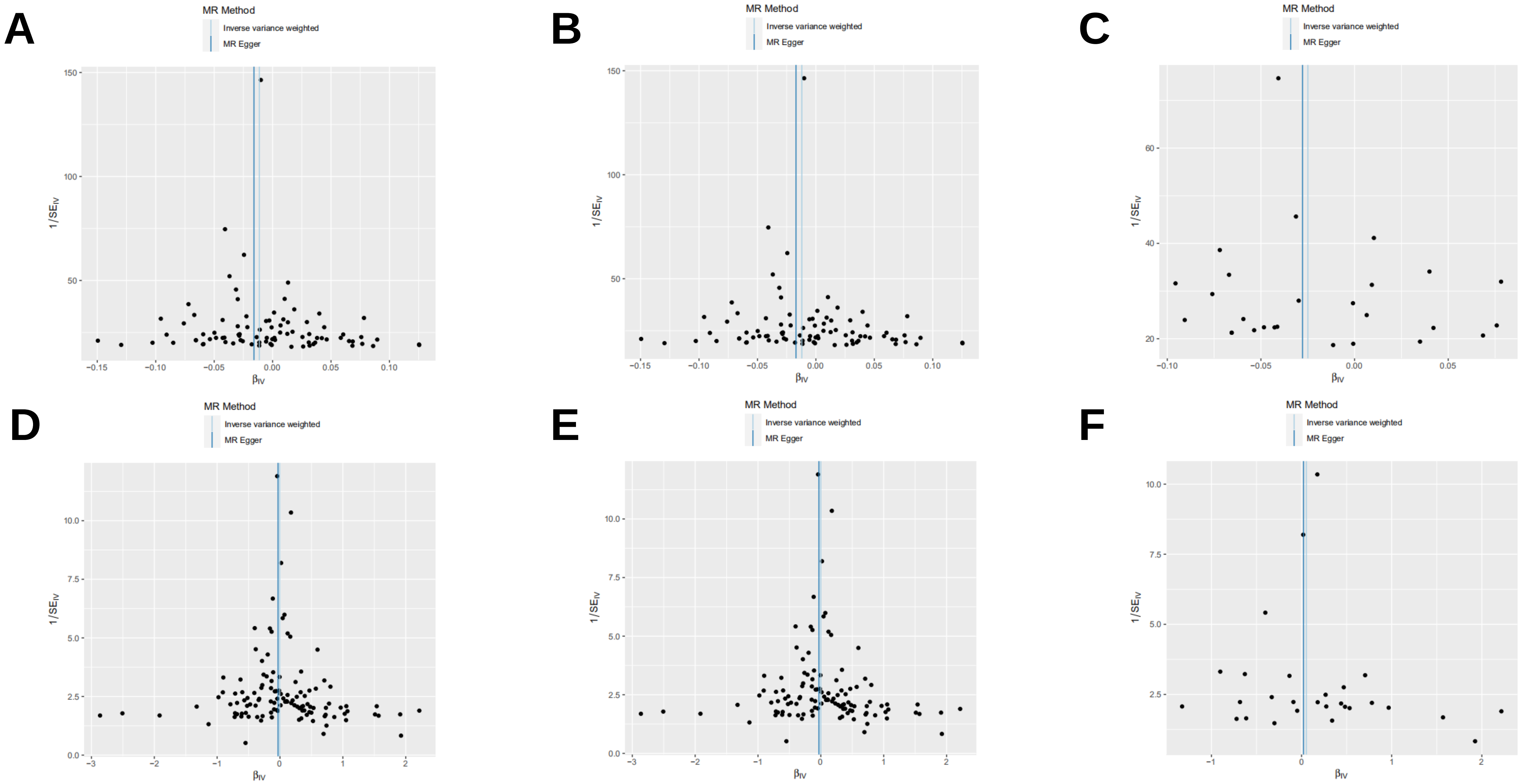

Supplementary Figure 3. Funnel plot to assess the robustness. A AF-LTL in MR analysis. B AF-LTL in eQTL-MR analysis. C AF-LTL in pQTL-MR analysis. D LTL-AF in MR analysis. E LTL-AF in eQTL-MR analysis. F LTL-AF in pQTL-MR analysis. The vertical lines represented the overall estimate obtained by the inverse variance weighted estimate and the MR-Egger regression. Scattering points denoted the effect estimated using a single SNP as an instrumental variable. LTL, leukocyte telomere length; AF, atrial fibrillation.
