# Supplementary material for: Causal relationship between atrial fibrillation and leukocyte telomere length: A two sample, bidirectional Mendelian randomization study
Source: Front Cardiovasc Med. 2023 Feb 15;10:1093255. doi: 10.3389/fcvm.2023.1093255 (PMC9975167; doi:10.3389/fcvm.2023.1093255)
Supplement: Supplementary file 8 [file Data_Sheet_8.PDF]

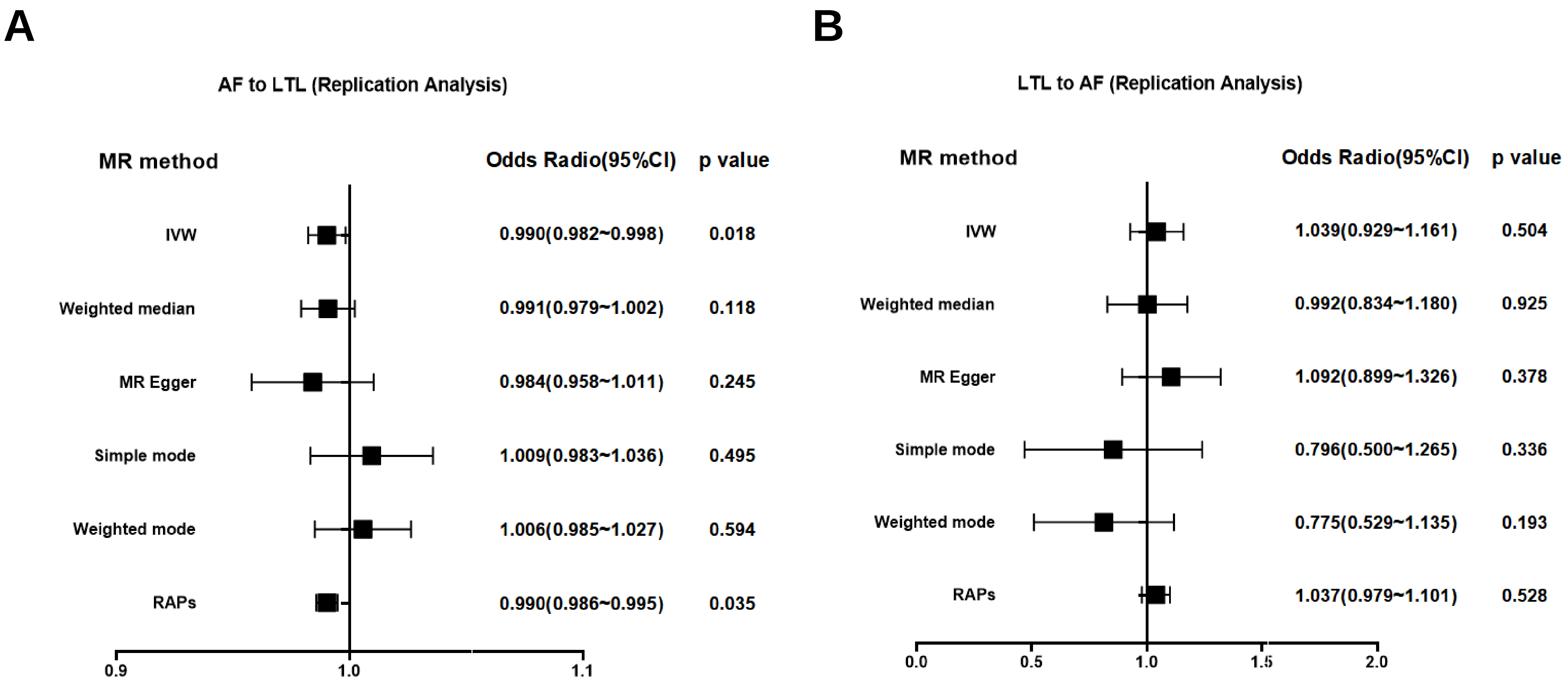

Supplementary Figure 4. Mendelian Randomization estimators of the causal relationship between leucocyte telomere length and atrial fibrillation in replication analyses. A AF-LTL. B LTL-AF. Analyses were conducted using the conventional IVW, Weighted median, MR Egger, Simple mode, Weighted mode, Robust adjusted profile score (RAPS) methods. The slope of each line corresponds to the estimated MR effect per method. MR, mendelian randomization; IVW, Inverse variance weighted; RAPS, robust adjusted profile score; LTL, leucocyte telomere length; AF, atrial fibrillation.
